# Age-Related Inflammation and Oxidative Stress in the Cochlea Are Exacerbated by Long-Term, Short-Duration Noise Stimulation

**DOI:** 10.3389/fnagi.2022.853320

**Published:** 2022-04-05

**Authors:** Verónica Fuentes-Santamaría, Juan Carlos Alvarado, Susana Mellado, Pedro Melgar-Rojas, María Cruz Gabaldón-Ull, José J. Cabanes-Sanchis, José M. Juiz

**Affiliations:** ^1^Instituto de Investigación en Discapacidades Neurológicas (IDINE), Albacete, Spain; ^2^Facultad de Medicina, Universidad de Castilla-La Mancha, Albacete, Spain; ^3^Department of Otolaryngology, Hannover Medical School, NIFE-VIANNA, Cluster of Excellence Hearing4all-German Research Foundation, Hanover, Germany

**Keywords:** accelerated presbycusis, age-related hearing loss, aging, antioxidants, inflammation

## Abstract

We have previously reported that young adult rats exposed to daily, short-duration noise for extended time periods, develop accelerated presbycusis starting at 6 months of age. Auditory aging is associated with progressive hearing loss, cell deterioration, dysregulation of the antioxidant defense system, and chronic inflammation, among others. To further characterize cellular and molecular mechanisms at the crossroads between noise and age-related hearing loss (ARHL), 3-month-old rats were exposed to a noise-accelerated presbycusis (NAP) protocol and tested at 6 and 16 months of age, using auditory brainstem responses, Real-Time Reverse Transcription-Quantitative PCR (RT-qPCR) and immunocytochemistry. Chronic noise-exposure leading to permanent auditory threshold shifts in 6-month-old rats, resulted in impaired sodium/potassium activity, degenerative changes in the lateral wall and spiral ganglion, increased lipid peroxidation, and sustained cochlear inflammation with advancing age. Additionally, at 6 months, noise-exposed rats showed significant increases in the gene expression of antioxidant enzymes (superoxide dismutase 1/2, glutathione peroxidase 1, and catalase) and inflammation-associated molecules [ionized calcium binding adaptor molecule 1, interleukin-1 beta (IL-1β), and tumor necrosis factor-alpha]. The levels of IL-1β were upregulated in the spiral ganglion and spiral ligament, particularly in type IV fibrocytes; these cells showed decreased levels of connective tissue growth factor and increased levels of 4-hydroxynonenal. These data provide functional, structural and molecular evidence that age-noise interaction contributes to exacerbating presbycusis in young rats by leading to progressive dysfunction and early degeneration of cochlear cells and structures. These findings contribute to a better understanding of NAP etiopathogenesis, which is essential as it affects the life quality of young adults worldwide.

## Introduction

Presbycusis or age-related hearing loss (ARHL) is a complex and multifactorial condition characterized by slow and progressive, auditory sensorineural degeneration, leading to hearing loss, progressing with advancing age (Fetoni et al., 2011; Keithley, 2019). Recent estimates for 2021 indicate that the prevalence of moderate to severe hearing loss in the aging population ranges globally from 15.4% among individuals aged 60–58.2% for those aged 90 or older (World Health Organization, 2021). The aging cochlea undergoes multiple regressive changes at the cellular and subcellular levels. These include loss of sensory cells, a decrease in the number of synaptic ribbons per inner hair cell, degeneration and death of spiral ligament fibrocytes and neurons, strial pathology, vascular dysfunction, and a decline in endocochlear potential, which together contribute to threshold elevation (Gratton et al., 1996; Gordon-Salant, 2005; Kidd and Bao, 2012; Viana et al., 2015; Parthasarathy and Kujawa, 2018; Keithley, 2019; Fischer et al., 2020; Chen et al., 2021). Human studies on the aging ear have extensively documented that damage to the sensory epithelium and the loss of cochlear neurons along with strial degeneration are common age-related features associated with presbycusis (Soucek et al., 1986; Schuknecht and Gacek, 1993; Nelson and Hinojosa, 2006). Indeed, evidence from post-mortem human temporal bones of aged individuals suggests that cochlear synaptopathy and primary cochlear neural degeneration, without significant inner hair cell loss, may also potentially contribute to this age-associated hearing disability (Viana et al., 2015; Wu et al., 2019).

Several animal models that exhibit different patterns of presbycusis-related progressive cochlear degeneration have been developed to investigate the pathogenesis of age-related cochlear damage and predict human disease (Fetoni et al., 2011; Alvarado et al., 2014; Bowl and Dawson, 2014; Escabi et al., 2019). The C57BL/6J (B6) mouse shows mixed sensory-neural-strial ARHL, which is characterized by progressive hearing loss for high frequencies that is already detectable at 6 months of age and extends to lower frequencies by 12 months. Additionally, these features are accompanied by degeneration of the lateral wall, spiral ganglion, and organ of Corti (Li and Borg, 1993; Di Girolamo et al., 2001; Carraro and Harrison, 2016). In contrast, CBA/CaJ mice have near-normal hearing, and exhibit a decline in endocochlear potential with aging, along with lateral wall atrophy and dysregulation of gene and protein expression of NKA subunits by 2 years of age (Sha et al., 2008, 2009; Ohlemiller et al., 2010; Hao et al., 2014; Ding et al., 2018). In rats, the Fischer 344 (F344) albino strain develops a progressive hearing deficit that initiates at 12 months of age, which is accompanied by strial pathology and outer hair cell loss (Buckiova et al., 2007; Bielefeld et al., 2008; Syka, 2010; Balogová et al., 2018). Similarly, Wistar rats also show age-related physiological alterations, including increased auditory thresholds, decreased wave amplitudes, and elongation of wave latencies that initiate at approximately 12–14 months of age and extend to 18–20 months of age; these rats also exhibit reduced excitatory and inhibitory neurotransmission in the aged cochlear nucleus (Alvarado et al., 2014).

Noise exposure may contribute to modify the onset and/or progression of auditory aging (Kujawa and Liberman, 2006; Alvarado et al., 2019; Wang and Puel, 2020). Valuable information has been obtained from reports evaluating the impact of a single noise exposure on aging in young CBA/CaJ mice (Kujawa and Liberman, 2006; Fernandez et al., 2015). However, humans are exposed daily for extended time periods to diverse and complex sources of noise, including urban, recreational, or occupational. Although studies reporting the effects of long-term daily noise exposure on hearing are limited, a previous study (Campo et al., 2011) using a noise exposure paradigm designed to mimic human responses, showed that moderate stimulation [8 kHz, 86.2 dB sound pressure level (SPL)] for 6 h per day, 5 days a week for 1 month in 6-month-old Norway rats accelerates ARHL. Consistent with this, our previous experiments using Wistar rats demonstrated a significant detrimental effect of long-term exposure to short-duration loud sound stimulation on young, 3-month-old animals [1 h of continuous white noise (110 dB SPL), 5 days a week] that were evaluated at 6, 12, and 18 months of age. The data showed that, at 6 months of age, these rats already had impaired auditory function and displayed physiological characteristics similar to those observed in 12-month-old unexposed rats, suggestive of a shortening of presbycusis onset (Alvarado et al., 2019).

Oxidative stress, with imbalances in the antioxidant defense system and adaptive and/or dysregulated inflammation are also crucial biological responses associated with ARHL and noise-induced hearing loss (NIHL) (Henderson et al., 2006; Tan, 2013; Okano, 2014; Kalinec et al., 2017; Fetoni et al., 2019). Key pathophysiological elements associated with the noise-exposed and aging cochlea include immune system dysregulation, reshaping of cytokine expression profiles, and dysregulation of redox homeostasis, including decreases in antioxidant protein and enzyme concentrations and increases in the levels of glutathione-conjugated proteins and the products of hydroxyl radical and peroxynitrite activity (Hirose et al., 2005; Fujioka et al., 2006; Tornabene et al., 2006; Jiang et al., 2007; Carroll et al., 2017; Hu et al., 2018; He et al., 2020; Lyu et al., 2020). As demonstrated in numerous studies on the aging and noise-exposed cochlea, the above-mentioned cellular processes are closely interlinked and are associated with detrimental effects on cochlear tissue and hearing (Nagashima et al., 2010; Fetoni et al., 2013, 2015b; Jayakody et al., 2018; Bermúdez-Muñoz et al., 2020). Despite the many studies that have addressed the link between age and noise, few have evaluated the chronic effect of noise exposure on the aging cochlea. Therefore, using a noise-accelerated presbycusis (NAP) protocol (Alvarado et al., 2019) and functional, histological, and molecular techniques, the aim of this study was to further evaluate the effects of long-term exposure to a short, loud noise (1 h of continuous white noise at 110 dB SPL) for 5 days beginning at 3 months and lasting until 16 months of age and assess which pathological and/or adaptive cellular processes might contribute to exacerbating the progression of presbycusis induced by chronic noise exposure.

## Materials and Methods

### Animals

For this study, 3-month-old adult male Wistar rats (*n* = 40) (Charles River, Barcelona, Spain) were used. The animals were housed and monitored at the University of Castilla-La Mancha animal facility (Albacete, Spain) under controlled conditions (temperature: 22–23°C; humidity: 60 ± 5%; 12:12 h light/dark cycle) and had *ad libitum* access to food and water. All procedures involving the use and care of the animals were approved by the Ethics Committee on Animal Experimentation at the University of Castilla-La Mancha (Permit Number: PR-2019-02-05) and conformed to European Union (Directive 2010/63/EU) and Spanish (R.D. 53/2013; Law 32/2007) regulations for the care and use of animals in research.

### Noise-Accelerated Presbycusis

The NAP protocol consisted of applying a repetitive, long-term, short-duration noise stimulation, as previously described (Alvarado et al., 2019). The stimulus was a continuous white noise presented at 110 dB SPL 1 h per day for 5 consecutive days, followed by 2 days of recovery before another stimulation cycle was initiated. Noise exposure was performed inside a reverberating chamber (methacrylate; 60 cm long × 70 cm wide × 40 cm high) with non-parallel and tilted walls to prevent standing waves and ensure a more homogeneous sound field. The chamber was located in a double-walled sound-attenuating booth placed within a sound-attenuating room. The NAP protocol started at 3 months of age and was repeated until the animals reached 16 months of age. Three-month-old rats were used as the control group (CTR, *n* = 8). For the experiments, rats were distributed into non-exposed (NE, *n* = 16) and noise-exposed (E, *n* = 16) groups, and then further divided into the following subgroups: (1) non-exposed, 6-month-old group (NE6); (2) noise-exposed, 6-month-old group (E6); (3) non-exposed, 16-month-old group (NE16); and (4) noise-exposed, 16-month-old group (E16). Half of the animals in each group were used for histology and half for Real-Time Reverse Transcription-Quantitative PCR (RT-qPCR).

### Physiological Assessment

#### Auditory Brainstem Response Recordings

Auditory function was tested at 3 months of age (CTR group; before the beginning the noise overexposure protocol), 6 months of age (NE6 and E6 groups), and 16 months of age (NE16 and E16 groups) using auditory brainstem response (ABR) recordings. Recordings in the exposed animals were performed after the recovery time and immediately before initiating the corresponding round of 5 days of noise stimulation or immediately before euthanasia. The procedure for the ABR recordings was performed as previously described (Alvarado et al., 2012, 2014, 2018, 2019, 2009; Fuentes-Santamaría et al., 2012, 2013, 2014; Melgar-Rojas et al., 2015). The animals were anesthetized with isoflurane administered *via* inhalation (1 L/min O_2_ flow rate) at the dose levels of 4% and 1.5–2% for induction and maintenance, respectively. After anesthesia, the rats were placed in a sound-attenuating and electrically shielded booth (EYMASA/INCOTRON S.L., Barcelona, Spain) that was placed inside a sound-attenuating room. Three subdermal needle electrodes (Rochester Electro-Medical, Tampa, FL, United States) were used, located at the vertex (non-inverting), the right mastoid (inverting), and the left mastoid (ground). Stimulation and recordings were performed using the BioSig System III (Tucker-Davis Technologies, Alachua, FL, United States). Before recording, calibration was performed using SigCal software (Tucker-Davis Technologies) and an ER-10B + low noise microphone system (Etymotic Research Inc., Elk Grove, IL, United States). Sounds generated digitally by the SigGenRP software and the RX6 Piranha Multifunction Processor hardware were delivered to the external auditory meatus of the right ear using an EDC1 electrostatic speaker driver (Tucker-Davis Technologies) through an EC-1 electrostatic speaker (Tucker-Davis Technologies). Pure tone burst sounds (5 ms rise/fall time without a plateau with a cos2 envelope delivered at 20/s) at seven different frequencies (0.5, 1, 2, 4, 8, 16, and 32 kHz) were used as auditory stimuli. Finally, evoked responses were filtered (0.3–3.0 kHz), averaged (500 waveforms), and stored for offline analysis.

### Auditory Brainstem Response Data Analysis

#### Auditory Thresholds

For all experiments, the maximum level of stimulus intensity was established at 80 dB to avoid any possible additional noise overstimulation trauma in the animals during the recordings (Gourévitch et al., 2009; Alvarado et al., 2012, 2014, 2018, 2019, 2009; Fuentes-Santamaría et al., 2012, 2013, 2014; Melgar-Rojas et al., 2015). For all frequencies evaluated, the background activity (before stimulus onset) and the evoked waves (after stimulus onset) were measured in 5 dB steps descending from 80 dB SPL. The auditory threshold was the stimulus intensity that evoked responses with a peak-to-peak voltage greater than 2 standard deviations (SD) of the background activity (Alvarado et al., 2012, 2014, 2016, 2018, 2019, 2009; Fuentes-Santamaría et al., 2012, 2013, 2014; Melgar-Rojas et al., 2015). For statistical purposes, if no auditory evoked responses were obtained at 80 dB, the thresholds were set at that value (Subramaniam et al., 1992; Trowe et al., 2008; Alvarado et al., 2012, 2014, 2018, 2019, 2009; Fuentes-Santamaría et al., 2012, 2013, 2014; Melgar-Rojas et al., 2015).

#### Threshold Shift

The threshold shift was also determined for each of the frequencies studied by subtracting the auditory thresholds at the different ages (6 and 16 months) from the auditory thresholds in the 3-month-old control rats (Alvarado et al., 2012, 2014, 2018, 2019, 2009; Fuentes-Santamaría et al., 2012, 2013, 2014; Melgar-Rojas et al., 2015).

### Histological Assessment

#### Immunohistochemistry

Rats were anesthetized (pentobarbital, 200 mg/kg) and perfused first with 0.9% saline and then with 4% paraformaldehyde solution in 0.1 M phosphate buffer (PB) (pH 7.3). Following dissection, left cochleae were postfixed in the same fixative solution, decalcified in 50% RDO rapid decalcifier solution (Apex Engineering Products Corporation, Aurora, IL, United States) for 2 h, cryoprotected (30% sucrose in 0.1 M PB), embedded (solution containing 15% sucrose and 10% gelatin), and cryosectioned through the modiolus at 20 μm. After several rinses in phosphate-buffered saline (PBS) containing 0.2% Triton X-100 (Tx), the sections were blocked for 1 h with 10% bovine serum albumin in PBS-Tx (0.2%), and then incubated overnight in a humidity box at 4°C with the following primary antibodies diluted in PBS-Tx (0.2%): mouse anti-Na^+^/K^+^-ATPase alpha-1 subunit (NKAα1), goat anti-connective tissue growth factor (CTGF), rabbit anti-4-hydroxynonenal (4-HNE), rabbit anti-ionized calcium-binding adaptor molecule 1 (Iba1), and goat anti-interleukin-1 beta (IL-1β) (see Table 1 for detailed information regarding each antibody). The next day, after extensive rinsing, the sections were incubated for 2 h with the corresponding biotinylated secondary antibody (1:200, Vector Laboratories, Burlingame, CA, United States) followed by incubation with an avidin–biotin–peroxidase complex solution (ABC kit, Vector Labs, Burlingame, CA, United States) for 1 h. To visualize the immunostaining, sections were exposed to 3,3’-diaminobenzidine tetrahydrochloride (DAB) solution, rinsed, and coverslipped using Cytoseal (Stephens Scientific, Kalamazoo, MI, United States). To avoid experimental artifacts, the following sets of control experiments were carried out to confirm the specificity of the immunohistochemistry detection system: (1) omission of the primary antibody by replacement with PBS-BSA; (2) omission of the secondary antibody; and (3) omission of ABC reagent. Immunostaining in cells and tissues was absent under these conditions. Immunostaining was assessed under a Nikon Eclipse 80i photomicroscope (Nikon Instruments Europe B.V., Amsterdam, Netherlands) using a 40 × objective. As previously described, images were captured using a DXM 1,200 C digital camera (Nikon Instruments Europe B.V., Amsterdam, Netherlands) attached to the microscope.

**TABLE 1:** Antibodies used for immunohistochemistry.

Primary antibody	Immunogen	Host	Code/clone	Dilution	Manufacturer
4-HNE	Free HNE-KLH coupled	Rabbit	HNE11-S	1:100	Alpha Diagnostic, San Antonio, Texas, United States
Iba1	C-terminus of Iba1′ (N′-PTGPPAKKAISELP-C′)	Rabbit	019-19741	1:2000	Wako Pure Chemical Industries, Neuss, Germany
NKAα1	Chicken NKAα1	Mouse	α6F	1:400	Developmental Studies Hybridoma Bank
CTGF	Synthetic peptide corresponding to Human CTGF	Goat	Ab238246	1:200	Abcam, Cambridge, MA, United States
IL-1β	C-terminus of rat IL-1β	Goat	SC-1252	1:100	Santa Cruz, Biotechnology, Inc. Germany

#### Fluorescent Double-Labeling

Right cochleae were processed for double-immunofluorescence labeling for CTGF/4-HNE or 4-HNE/Phalloidin or Iba1/IL-1β (Table 1). After incubation with the respective primary antibodies (as described in the previous section), the sections were subjected to four 15-min washes in Tris-buffered saline (TBS)-Tx (0.2%), and then incubated with fluorescently labeled secondary antibodies (1:200, Molecular Probes, Eugene, OR, United States) and fluorescently labeled phalloidin (Pha) for 2 h at room temperature. After counterstaining with DAPI, the sections were coverslipped and kept overnight at 4°C. Immunofluorescence was examined with a multichannel laser scanning confocal microscope (LSM 710; Zeiss, Germany) (excitation wavelengths: 405, 488, and 594 nm). Thin optical sections (2.5 μm) were acquired for each dye using a 40x Plan Apo oil-immersion objective (1.4 NA) and stacked to create high-resolution Z-series with standardized parameters including for camera gain, pinhole size, laser intensity, tissue thickness, and optical sectioning. Maximum intensity projections of Z-stacks were obtained using ZEN 2009 Light Edition software (Zeiss).

### Data Analysis

#### Measurements of Cross-Sectional Areas and Counting of Spiral Ganglion Neurons

As previously described (Fuentes-Santamaría et al. 2006, 2014; Shin et al., 2019), the morphometric analysis of the spiral ganglion was performed on Nissl-stained mid-modiolar cochlear sections (20 μm apart; four sections per animal, 4 animals per group) and only neurons with a well-delineated cytoplasm and visible nucleus and nucleolus were included in the analysis. Images of selected fields (40x magnification) were captured, and the number of neurons/field and their cross-sectional areas measured by using ImageJ software (NIH, Bethesda, MD, United States). Data were normalized relative to the control condition, and expressed as percentages ± SEM.

#### Measurements of Stria Vascularis Thickness

The analysis was performed on Nissl-stained mid-modiolar cochlear sections (20 μm apart; four sections per animal, 4 animals per group, as previously reported (Engle et al., 2013; Ding et al., 2018; Shin et al., 2019). Images of selected fields (40x magnification) were captured, and the SV thickness measured by using ImageJ software (NIH, Bethesda, MD, United States). The SV of each cochlea was divided into 10 orthogonal measurements along its length and the values obtained were averaged, normalized relative to the control condition, and expressed as percentages ± SEM.

#### Evaluation of Immunostaining

To evaluate differences in immunostaining across animal groups and corroborate the qualitative immunohistochemical data, a densitometric analysis of mid-modiolar cochlear sections (20 μm apart; four sections per animal, 4 animals per group) stained with NKAα1, 4-HNE, CTGC, and IL-1β antibodies was performed using ImageJ software (NIH, Bethesda, MD, United States). Fluorescence images of selected fields (40x magnification) were captured with a confocal laser-scanning microscope from tissue of interest, including the spiral ligament, the spiral limbus, and the spiral ganglion of unexposed and noise-exposed young adult and aged rats. As previously (Herborn et al., 2002; Fuentes-Santamaría et al., 2003, 2005, 2007a,b; Alvarado et al., 2004, 2009; Stanojlovic et al., 2019; Wang et al., 2020), for comparisons among animal groups, images were normalized and thresholded and the mean pixel intensity for each marker in each cochlear region for each animal group was assessed by evaluating the mean gray level of the immunostaining, as an indirect measure of protein content, and the percentage of variation of the immunostaining (expressed as a percentage of increase/decrease relative to the control condition (Yao and Godfrey, 1997; Lin and Talman, 2000; Fuentes-Santamaría et al., 2019).

### Cochlear Dissection and RNA Extraction

For tissue collection, animals were deeply anesthetized with 1.5–2% isoflurane (1 L/min O2 flow rate) (Esteve Pharmaceuticals, Barcelona, Spain) followed by an intraperitoneal injection of a combination of ketamine (80 mg/kg) (Pfizer Inc., New York, NY, United States) and xylazine (10 mg/kg) (Calier, S.A., Barcelona, Spain). After euthanasia, the whole cochlea was dissected from the temporal bone, rapidly frozen on dry ice, and stored at –80°C. Tissue was lysed using cold TRIzol reagent (Invitrogen, Thermo Fisher Scientific, Waltham, MA United States) following the manufacturer’s instructions for total RNA extraction. Cochleae were homogenized using a Polytron PT2100 homogenizer (Kinematica, dispersing aggregate PT-DA 2105/2EC; Rotor–Ø: 3 mm) at 30,000 rpm for < 30 s. Total RNA quantity and quality were determined by electrophotometry (NanoDrop ND-1000, Thermo Fisher Scientific) and gel electrophoresis. All RNA samples showed suitable A260/A280 and 28S/18S ratios.

### Real-Time Reverse Transcription-Quantitative PCR

Total RNA (1 μg) was reverse transcribed into cDNA using the RevertAid First Strand cDNA Synthesis Kit (Thermo Fisher Scientific). The reaction conditions were as previously published (Melgar-Rojas et al., 2015). cDNA was diluted 1:10 for qPCR, which was performed in a One Step Plus Real-Time PCR System (Applied Biosystems, Thermo Fisher Scientific) using Fast SYBR Green Master Mix (Thermo Fisher Scientific). Forward and reverse primers were used at a final concentration of 0.1 or 0.5 μM. Glyceraldehyde-3-phosphate dehydrogenase (GAPDH) was used as an internal control. The primer sequences are detailed in Table 2. The amplification efficiency was calculated as: Efficiency (%) = [–1 + 10 (–1/slope)] × 100. All qPCR runs included non-template controls (NTCs) that generated Cq values > 35. The experiments were performed in technical triplicates. Quantification of expression (fold change) from the Cq data was calculated using Step One Software v2.3 (Applied Biosystems) using the ΔΔCq method (Schmittgen and Livak, 2008). After obtaining the ΔCq, the ΔΔCq of each gene of interest was calculated as: ΔCq (noise-exposed group) –ΔCq (control group). Relative expression (fold change) was calculated as 2–ΔΔCq. All qPCR experiments complied with Minimum Information for Publication of Quantitative Real-Time PCR Experiments (MIQE) guidelines (Bustin et al., 2009). The percentage of variation of the relative expression (expressed as a percentage of increase/decrease relative to the NE6 condition) of each gene was also calculated.

**TABLE 2:** Oligonucleotides and qPCR settings.

Gene	GeneBank accession number	Primer sequence (5’–3’)	Genomic location (exons; FW-RV)	Product size (bp)	PCR efficiency	Regression coefficient (*R*^2^)	Final concentration
*Cat*	NM_012520	FW: GAGGAAACGCCTGTGTGAGA RV: TTGGCAGCTATGTGAGAGCC	11–13	201	98.8 %	0.9997	0.1 μM
*Gapdh*	NM_017008	FW: AGACAGCCGCATCTTCTTGT RV: CTTGCCGTGGGTAGAGTCAT	1–3	207	90.9 %	0.9975	0.1 μM
*Gpx1*	NM_030826	FW: GTTTCCCGTGCAATCAGTTC RV: CATTCCGCAGGAAGGTAAAG	1–2	71	99.3 %	0.9972	0.5 μM
*Iba1*	NM_017196	FW: TGCTGAAAGCCCAACAGGAA RV: CGTCTTGAAGGCCTCCAGTT	3–3/4*	113	90.8%	0.9891	0.1 μM
*IL1-β*	NM_031512	FW: AGCTTTCGACAGTGAGGAGAA RV: TCATCTGGACAGCCCAAGTC	2/3–4	99	97.7 %	0.9984	0.5 μM
*Sod1*	NM_017050	FW: CCACTGCAGGACCTCATTTT RV: CACCTTTGCCCAAGTCATCT	3–5	216	99.1 %	0.9991	0.1 μM
*Sod2*	NM_017051	FW: CTGGACAAACCTGAGCCCTA RV: GACCCAAAGTCACGCTTGATA	3–4	77	92.4 %	0.9935	0.1 μM
*TNF-α*	NM_012675	FW: GAGAAGTTCCCAAATGGGCT RV: TTGCTACGACGTGGGCTACG	1/2–3/4	109	102.6 %	0.9853	0.1 μM

*Primer that match on an exon–exon junction.

### Statistics

Unless otherwise specified, all data are presented as means ± SEM. Wave amplitudes and latencies were measured at 80 dB SPL and comparisons among the different groups were performed using two-way repeated-measures analysis of variance (ANOVA). Noise exposure (non-exposed vs. exposed) was used as an independent variable and age (3, 6, and 16 months) as a repeated independent variable. The ABR parameters and densitometric measures were dependent variables. For each frequency studied, the possible statistically significant main effect of age and noise exposure on each dependent variable time was evaluated. If the analysis showed a significant effect or interaction of the variables, a Scheffé *post hoc* analysis was used for comparisons among groups. Significance levels (α) and power (β) were set to 0.05 and 95%, respectively. Figures were prepared using the Canvas (Deneba v6.0) software package while graphs and statistics were generated by MS Excel and Statistica software, respectively.

## Results

### Effects of Repeated, Long-Term Exposure to Short-Duration Noise Stimulation on Aging

**FIGURE 1:**
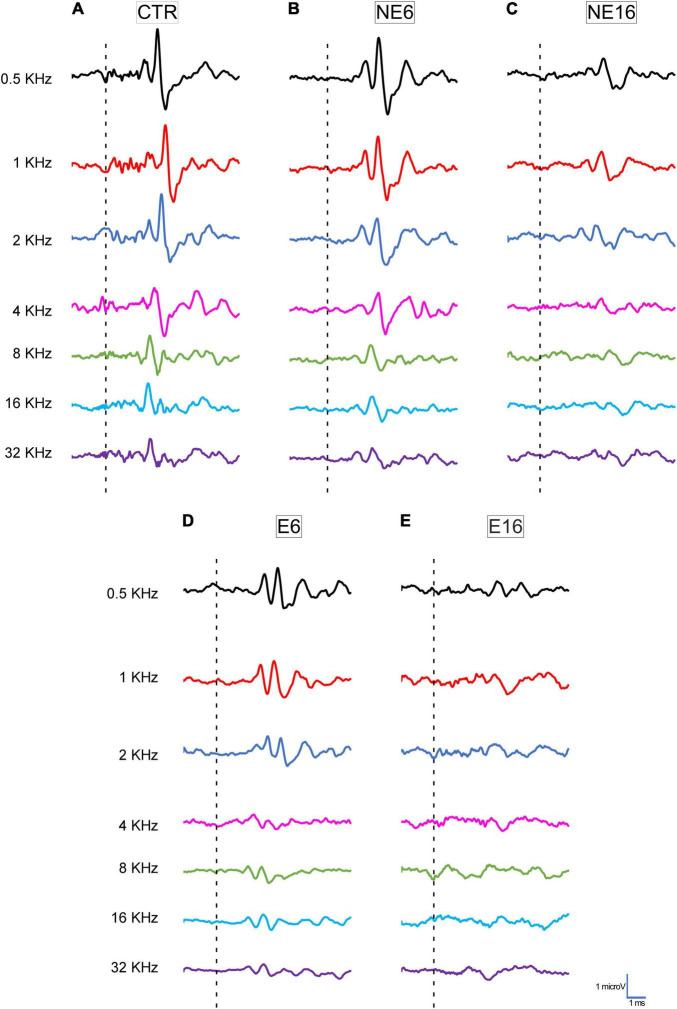
Line graphics of representative ABR traces from 3-month-old control rats (CTR) and from non-exposed (NE6 and NE16) and NAP-exposed (E6 and E16) rats for all frequencies tested. CTR **(A)** and NE6 **(B)** rats presented a similar recording pattern with the distinctive 4–5 waveforms after stimulus onset. In NE16 **(C)** rats, there was a characteristic decrease in the amplitude of all waves for all frequencies evaluated. In NAP-exposed rats, reduced wave amplitude was already detected at six months of age **(D)** and was more profound after 16 months **(E)** compared to CTR and non-exposed animals **(A,B)**. Dashed lines indicate stimulus onset. Stimulus intensity = 80 dB SPL; *n* = 4 for each animal group.

Typical ABR traces of unexposed and NAP-exposed rats are depicted in Figure 1. Consistent with previous studies on Wistar rats (Overbeck and Church, 1992; Church et al., 2010, 2012a,b; Alvarado et al., 2012, 2014, 2018), increasing age (NE16) led to a reduction in the amplitudes of all five waves evaluated at all frequencies when compared with younger (CTR and NE6) rats (Figures 1A–C; Alvarado et al., 2014, 2018, 2019). Following the NAP protocol, compared with unexposed animals (CTR, NE6, and NE16 groups; Figures 1A–C), decreased wave amplitudes were already noticeable at 6 months of age (E6, Figure 1D) and persisted in 16-month-old rats (E16, Figure 1E).

**FIGURE 2:**
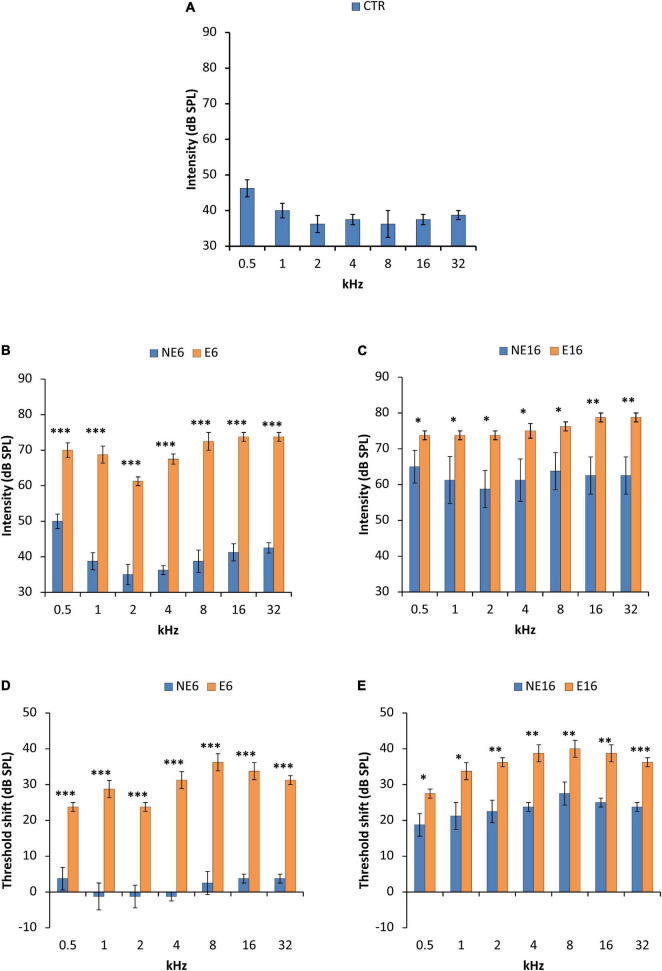
Bar graphs illustrating auditory thresholds and threshold shifts in 3-month-old control rats (CTR) and in non-exposed (NE6 and NE16) and NAP-exposed (E6 and E16) rats for all frequencies assessed. The mean values found in CTR **(A)** and NE6 (blue bars in **B**) rats were similar, with threshold shifts (blue bars in **D**) between –1.3 and + 3.8 relative to CTR animals. However, as animals aged (NE16), the mean thresholds (blue bars in **C**) and threshold shifts (blue bars in **E**) were higher than in CTR and NE6 rats. Following NAP, mean threshold values (orange bars in **B**) and threshold shifts (orange bars in **D**) were elevated as early as 6 months of age and were significantly different from those in CTR and NE6 rats. The oldest noise-exposed rats presented even higher thresholds (orange bars in **C**) and threshold shifts (orange bars in **E**) relative to CTR and unexposed animals. *p < 0.05; ^**^p < 0.01; ^***^p < 0.001. *n* = 4 for each animal group.

Regarding the auditory thresholds in unexposed animals, the mean values obtained for young (CTR, blue bars in Figure 2A and Table 3), adult (NE6, blue bars in Figure 2B and Table 3), and old (NE16, blue bars in Figure 2C and Table 3) rats were similar to those previously reported for Wistar rats. Auditory thresholds increased significantly with age (Jamesdaniel et al., 2008; Alvarado et al., 2012, 2014, 2018, 2019, 2009; Fuentes-Santamaría et al., 2012, 2013, 2014; Pilati et al., 2012; Melgar-Rojas et al., 2015). When compared with 3-month-old, unexposed control rats, 6-month-old rats showed threshold shifts ranging from –1.3 to 3.8 dB SPL, which were not statistically significant (NE6, blue bars in Figure 2D and Table 3). At 16 months (NE16, blue bars in Figure 2E and Table 3) threshold shifts reached statistically significant values of 18.8–27.5 dB SPL, thus confirming age-related threshold increases (Alvarado et al., 2014, 2018, 2019). In response to noise exposure, the auditory thresholds at 6 (E6, orange bars in Figure 2B and Table 3) and particularly at 16 (E16, orange bars in Figure 2C and Table 3) months of age, were significantly elevated at all frequencies tested when compared with those of age-matched NE and CTR rats. The average threshold shifts relative to CTR animals ranged from 23.8 to 36.3 dB SPL in the E6 group (orange bars in Figure 2D and Table 3) and from 27.5 to 40.0 dB SPL in the E16 group (orange bars in Figure 2E and Table 3), suggesting that noise interacts with auditory aging to further reduce hearing sensitivity.

**TABLE 3:** Mean, SE, and ANOVA analysis of the interaction between age and noise exposure over the thresholds and the threshold shifts.

		Frequencies (kHz)						
		0.5	1	2	4	8	16	32
**Threshold**	**(1)**	46.3 ± 2.4	40.0 ± 2.0	36.3 ± 2.4	37.5 ± 1.4	36.3 ± 3.8	37.5 ± 1.4	38.8 ± 1.3
	**(2)**	50.0 ± 2.0	38.8 ± 2.4	35.0 ± 2.9	36.3 ± 1.3	38.8 ± 3.1	41.3 ± 2.4	42.5 ± 1.4
	**(3)**	65.0 ± 4.6	61.3 ± 6.6	58.8 ± 5.2	61.3 ± 5.9	63.8 ± 5.2	62.5 ± 5.2	62.5 ± 5.2
	**(4)**	70.0 ± 2.0	68.8 ± 2.4	61.3 ± 1.3	67.5 ± 1.4	72.5 ± 2.5	73.8 ± 1.3	73.8 ± 1.3
	**(5)**	73.8 ± 1.3	73.8 ± 1.3	73.8 ± 1.3	75.0 ± 2.0	76.3 ± 1.3	78.8 ± 1.3	78.8 ± 1.3
	***F*_(3, 52_**_)=_	15.9 (***)	18.5 (***)	136.9 (***)	85.0 (***)	31.7 (***)	68.6 (***)	145.6 (***)

		**0.5**	**1**	**2**	**4**	**8**	**16**	**32**

**Threshold shift**	**(2)**	3.8 ± 3.1	–1.3 ± 3.8	–1.3 ± 3.1	–1.3 ± 1.3	2.5 ± 3.2	3.8 ± 1.3	3.8 ± 1.3
	**(3)**	18.8 ± 5.2	21.3 ± 8.0	22.5 ± 6.0	23.8 ± 5.5	27.5 ± 7.2	25.0 ± 5.4	23.8 ± 4.3
	**(4)**	23.8 ± 1.3	28.8 ± 2.4	23.8 ± 1.3	31.3 ± 2.4	36.3 ± 2.4	33.8 ± 2.4	31.3 ± 1.3
	**(5)**	27.5 ± 1.4	33.8 ± 3.1	36.3 ± 1.3	38.8 ± 3.8	40.0 ± 2.0	38.8 ± 1.3	36.3 ± 1.3
	***F*_(2, 53)=_**	15.4 (***)	20.8 (***)	37.8 (***)	74.1 (***)	29.6 (***)	67.8 (***)	228.4 (***)

**(1)** CTR; **(2)** NE6; **(3)** NE16; **(4)** E6; **(5)** E16; ***p < 0.001.

The statistical significance of the effects of age and noise exposure on auditory thresholds and threshold shifts was shown by ANOVA [*F*(3, 52) = 162.23, *p* < 0.0001 for absolute auditory thresholds and *F*(2, 53) = 185.24, *p* < 0.0001, for threshold shifts]. Scheffé *post hoc* test revealed no differences in threshold values between 3-month-old (CTR) and 6-month-old (NE6) animals; however, NE16 animals showed a significant increase in auditory thresholds and threshold shifts at all frequencies as a function of age (blue bars in Figures 2C,E and Table 3), which was consistent with previous findings (Alvarado et al., 2014, 2018, 2019). Scheffé *post hoc* test (Table 3) also showed that the auditory thresholds and threshold shifts were significantly increased in noise-exposed rats at all frequencies, both at 6 (E6, orange bars in Figures 2B,D) and 16 (E16, orange bars in Figures 2C,E) months of age when compared with those of age-matched, non-exposed animals (NE6 and NE16; blue bars in Figures 2B–E). These results reaffirmed the interaction of both age and noise with auditory thresholds and threshold shifts.

### Cochlear Histopathology Associated With Age-Related Hearing Loss and Noise-Accelerated Presbycusis

#### Structural Alterations in the Cochlea

**FIGURE 3:**
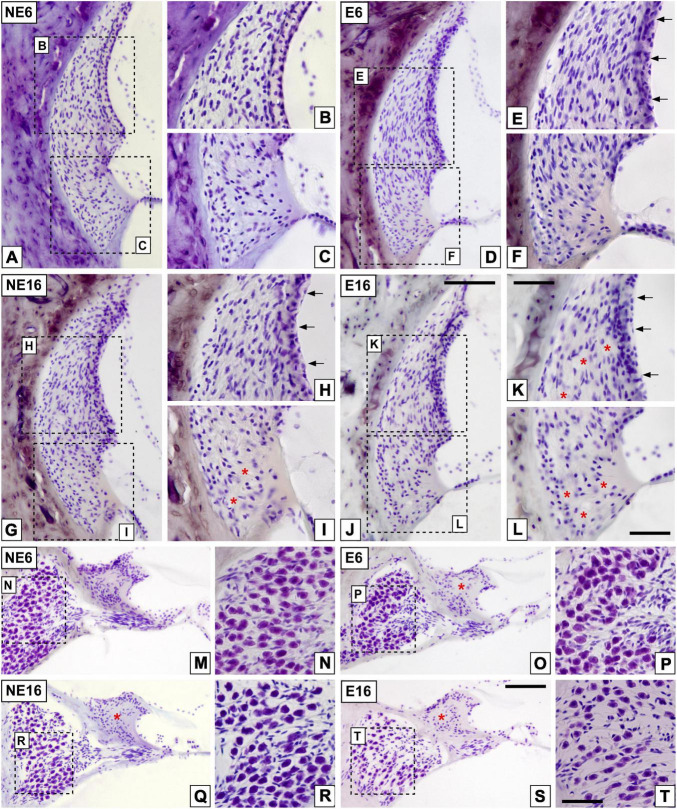
Digital images illustrating cochlear histopathology in NE6, E6, NE16, and E16 rats. **(A–L)** Cochlear mid-modiolar sections showing the spiral ligament in unexposed **(A–C)** and noise-exposed **(D–F)** young rats and unexposed **(G–I)** and noise-exposed **(J–L)** aging rats. Notice the abnormal stria morphology in E6, NE16, and E16 rats (arrows in **E,H,K**) compared to that in unexposed younger rats **(B)**. Loss of fibrocytes integrity in NE16 (* in **I**) and E16 (* in **K,L**) cochleae was also noticeable relative to the NE6 **(A–C)** and E6 **(D–F)** groups. **(M–T)** Cochlear mid-modiolar sections showing the spiral limbus and the spiral ganglion in unexposed **(M,N)** and NAP-exposed adult **(O,P)** rats and unexposed **(Q,R)** and NAP-exposed aging **(S,T)** rats. Missing spiral limbus cells were detected in the E6, NE16, and E16 groups (* in **O,Q,S**) while degeneration of spiral ganglion neurons was seen only in the oldest noise-exposed group **(S,T)**. Dashed square boxes in low-magnification images in **(A,D,G,J)** indicate the approximate locations of the higher-magnification images shown for each group. Arrows indicate strial dystrophy and asterisks indicate missing cells in each cochlear structure. Scales bars: 100 μm in **(J,S)**; 50 μm in **(K,L,T)**. *n* = 4 for each animal group.

To determine histological correlates of ARHL and NAP, the cochleae of young/adult and aged rats exposed or not to noise was examined, using cryostat sections stained with the Nissl method (Figure 3). Compared with non-exposed animals (NE6, Figures 3A–C), noise-exposed rats of the same age (E6, Figures 3D–F) exhibited early dystrophic changes in the SV, including an apparent disorganization of basal, intermediate, and marginal cell layers (arrows in Figure 3E). The lateral wall of aged (NE16) rats also displayed similar structural damage in the SV as well as presumptive type IV fibrocyte loss “patches” in the spiral ligament (Figure 3G, arrows and asterisks in Figures 3H–I) when compared to adult noise-exposed (E6) animals (Figures 3D–F), but not with that of adult, non-exposed rats (Figures 3A–C). These degenerative, age-related changes in the cochlear lateral wall were accelerated by repeated, long-term overexposure to a noise of short duration (E16), resulting in severe strial dystrophy and a greater extent of fibrocyte loss (Figure 3J, arrows and asterisks in Figures 3K,L). Note the increased gaps between cells in the stria vascularis (SV) of aged rats exposed to noise (arrows in Figure 3K) as compared to the other animal groups.

Quantitative analyses supporting these structural cochlear alterations showed increased thickness in the SV of unexposed and noise-exposed aging animals (NE16 and E16) when compared to unexposed and exposed (E6) younger animals (Table 4; see also Figure 3). Note that such increases were evident in all cochlear turns and were about 18 and 24% for NE16 and E16; respectively.

**TABLE 4:** Mean, SE, and ANOVA analysis of the interaction between age, noise over exposure and stria vascularis thickness in the cochlea relative to the control condition.

	Basal	Middle	Apical
**CTR**	100.00 ± 0.00	100.00 ± 0.00	100.00 ± 0.00
**E6**	102.04 ± 1.35	102.54 ± 1.26	100.93 ± 1.27
**NE16**	117.69 ± 1.31	118.78 ± 1.48	115.46 ± 1.26
**E16**	123.68 ± 1.45	121.33 ± 1.39	122.00 ± 1.61
	***F*_(3, 116_**_)_ = 19.85 (***)	***F*_(3, 116_**_)_ = 15.72 (***)	***F*_(3, 116_**_)_ = 17.43 (***)

Significance levels				
***Basal***	**CTR**	**E6**	**NE16**	**E16**
**CTR**		NS	***	***
**E6**	NS		***	***
**NE16**	***	***		NS
**E16**	***	***	NS	
***Middle***	**CTR**	**E6**	**NE16**	**E16**
**CTR**		NS	***	***
**E6**	NS		**	***
**NE16**	***	**		NS
**E16**	***	***	NS	
***Apical***	**CTR**	**E6**	**NE16**	**E16**
**CTR**		NS	***	***
**E6**	NS		**	***
**NE16**	***	**		NS
**E16**	***	***	NS	

**p < 0.01, ***p < 0.001.

*NS, Not Significant.*

Additionally, NAP resulted in the loss of the interdental cells of the spiral limbus in E6 (asterisk in Figure 3O), NE16 (asterisk in Figure 3Q), and E16 (asterisk in Figure 3S) rats and an apparent loss of SGN in NE16 (Figures 3Q,R), E16 (Figures 3S,T) rats but not in rats of the NE6 (Figures 3M,N) and E6 (Figures 3O,P) groups. Quantification of these degenerative changes in the spiral ganglion showed a significant age-associated decrease in spiral ganglion cells survival that was more noticeable in the basal and middle turns than in the apical turn relative to the control condition (Table 5; see also Figure 3). In addition, to evaluate possible neuronal atrophy associated to noise-exposure, aging or both, the cross-sectional areas of SG neurons were evaluated for each condition. As shown in Table 6, the average cell areas for each frequency region were not significantly different among groups.

**TABLE 5:** Mean, SE, and ANOVA analysis of the interaction between age, noise over exposure and spiral ganglion cells survival in the cochlea relative to the control condition.

	Basal	Middle	Apical
**CTR**	100.00 ± 0.00	100.00 ± 0.00	100.00 ± 0.00
**E6**	96.02 ± 1.19	96.88 ± 0.99	97.89 ± 1.04
**NE16**	72.87 ± 0.93	82.81 ± 1.05	81.00 ± 1.16
**E16**	36.24 ± 1.12	67.19 ± 1.12	75.43 ± 1.11
	***F*_(3,36_**_)_ = 199.03 (***)	***F*_(3,36_**_)_ = 105.23 (***)	***F*_(3,36_**_)_ = 36.20 (***)

Significance levels				
***Basal***	**CTR**	**E6**	**NE16**	**E16**
**CTR**		NS	***	***
**E6**	NS		***	***
**NE16**	***	***		***
**E16**	***	***	***	
***Middle***	**CTR**	**E6**	**NE16**	**E16**
**CTR**		NS	***	***
**E6**	NS		***	***
**NE16**	***	***		***
**E16**	***	***	***	
***Apical***	**CTR**	**E6**	**NE16**	**E16**
**CTR**		NS	***	***
**E6**	NS		***	***
**NE16**	***	***		NS
**E16**	***	***	NS	

***p < 0.001.

*NS, Not Significant.*

**TABLE 6:** Mean, SE, and ANOVA analysis of the interaction between age, noise over exposure and cross-sectional areas of spiral ganglion cells in the cochlea relative to the control condition.

	Basal	Middle	Apical
**CTR**	100.00 ± 0.00	100.00 ± 0.00	100.00 ± 0.00
**E6**	98.91 ± 1.75	102.33 ± 1.46	99.60 ± 1.43
**NE16**	102.99 ± 1.83	98.62 ± 1.43	102.42 ± 1.59
**E16**	101.62 ± 2.08	102.70 ± 1.67	100.90 ± 1.51
	***F*_(3, 156_**_)_ = 0.86 (NS)	***F*_(3, 239_**_)_ = 1.52 (NS)	***F*_(3, 180_**_)_ = 0.51 (NS)

*NS, Not significant.*

#### Dysregulation of NKAα1

**FIGURE 4:**
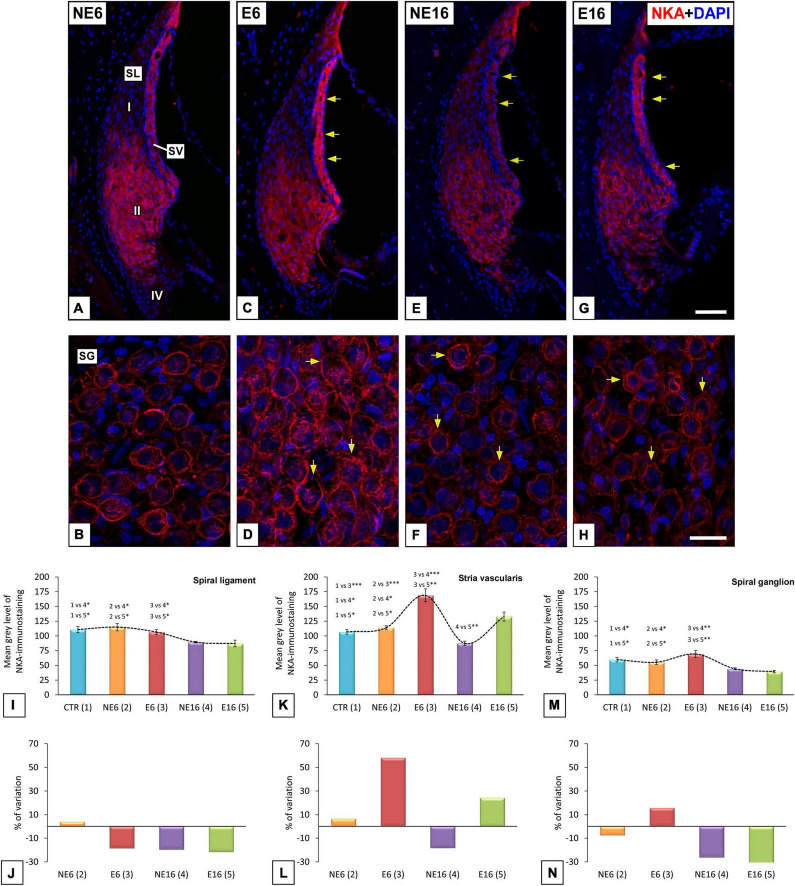
Digital images illustrating NKA-immunostaining in NE6, E6, NE16, and E16 rats. Noise exposure in adult (E6) rats leads to increased staining levels in SV (arrows in **C**), and SG (arrows in **D**) compared to NE6 **(A,B)**, NE16 (arrows in **E,F**) and E16 (arrows in **G,H**) rats. Note, however, that staining levels in the SL and SG of aged cochleae (arrows in **E–H**) were decreased relative to young **(A–D)** rats. The quantification of the mean gray levels of NKA-staining **(I–M)** and the percentage of variation **(J–N)** for each cochlear structure and animal group confirmed the histological data. Cell nuclei (blue) were counterstained with DAPI.SL, spiral ligament; SV, stria vascularis. *p < 0.05; ^**^p < 0.01; ^***^p < 0.001. Scales bars: 50 μm in **(G)**; 20 μm in **(H)**. *n* = 4 for each animal group.

To further evaluate cochlear dysfunction in response to the combined effect of aging and NAP, the cellular distribution and location of NKAα1, an ion transport protein involved in the regulation of the endocochlear potential generated by the SV (Schulte and Adams, 1989; Cate et al., 1994; McLean et al., 2009; Yamaguchi et al., 2014), were assessed in lateral wall structures and spiral ganglions of cochleae from young/adult and aged rats exposed or not to noise (Figure 4). NKAα1 staining in NE6 rats was localized to the SV, type II spiral ligament fibrocytes (Figure 4A), and the spiral ganglion (Figure 4B). NKAα1 immunostaining in noise-exposed adult (E6) rats was stronger in the SV (arrows in Figure 4C) and spiral ganglion neurons (SGN) (arrows in Figure 4D) relative to that of unexposed younger animals (NE6; Figures 4A,B). In older rats of the NE16 and E16 groups, NKAα1 staining declined in the lateral wall and the spiral ganglion (Figures 4E–H) when compared with that of rats in the E6 and NE6 groups; however, staining was stronger in the SV (arrows in Figure 4G), but not the spiral ganglion (arrows in Figure 4H) of E16 rats compared with that in age-matched NE16 animals (arrows in Figures 4E,F). Even though pathological alterations were observed in the cochlear microvasculature of E6, NE16, and E16 rats (arrows in Figures 4C,E,G), strial capillary damage was more extensive in the rats of the E16 group (Figure 4G). These findings are compatible with a possible interaction between age and noise affecting NKAα1 function in the inner ear.

These observations were confirmed by ANOVA, which showed a significant effect of both age and noise exposure on the mean gray values for NKAα1 in the spiral ligament [*F*(4, 24) = 7.12, *p* < 0.001], SV [*F*(4, 27) = 24.43, *p* < 0.001], and spiral ganglion [*F*(4, 29) = 6.96, *p* < 0.001]. Further analysis using the Scheffé *post hoc* test indicated that the mean gray value for NKAα1 in the spiral ligament was significantly decreased in both the NE16 and E16 groups (–19.41% and –21.47%, respectively) when compared to CTR as well as the NE6 and E6 groups (Figures 4I,J). Regarding the SV, the mean gray value for NKAα1 was significantly increased in E6 animals (+ 58.17%) compared with that in the other groups (Figures 4K,L). Notably, although the mean gray value for NKAα1 was higher in the E16 group than in the NE16 group (*p* < 0.05), which suggested that the increase was associated with noise overexposure, the difference did not reach significance when compared with those in CTR and NE6 rats. In the spiral ganglion, there was a significant reduction in the mean gray values for NKAα1 in the NE16 and E16 rats (–26.10 and –34.10%, respectively) compared with that in the other groups (Figures 4M,N).

#### Susceptibility of CTGC-Positive Type IV Fibrocytes to Age and Noise Exposure

**FIGURE 5:**
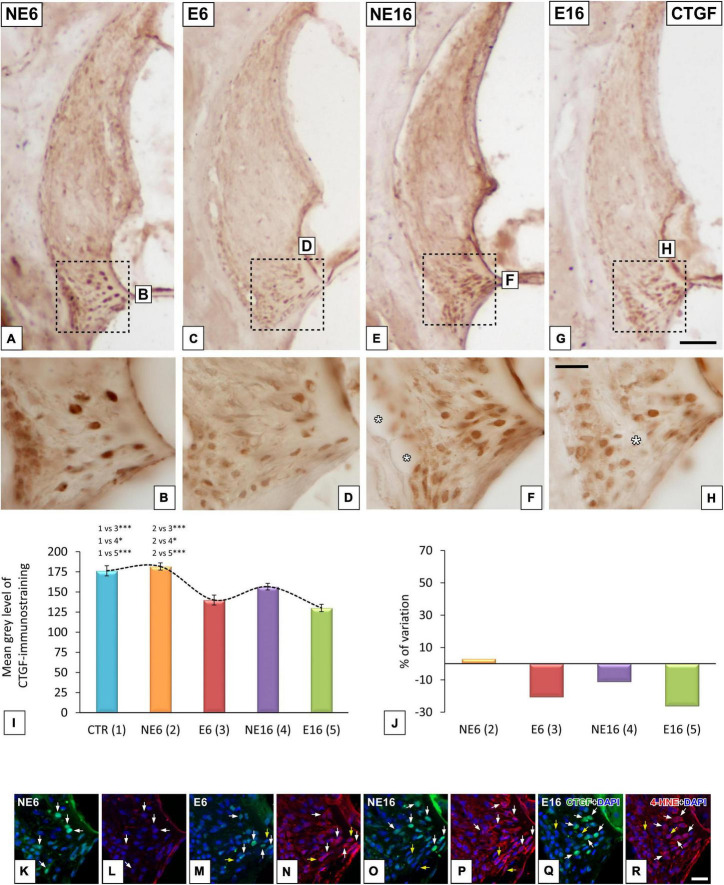
Digital images illustrating CTGF-immunostained type IV fibrocytes in the spiral ligaments of young/adult and aging rats exposed to NAP. NAP-exposure in E6 **(C,D)** and E16 **(J,G,H)** rats resulted in decreased CTGF staining relative to unexposed control rats **(A,B)**. Note, however, that in the aging unexposed group **(E,F)** these levels were increased relative to the E6 and E16 groups. The quantification of the immunostaining corroborated these qualitative results **(I,J)**. Double immunohistochemical staining for CTGF (green) and 4HNE (red) in the spiral ligaments of E6, NE16, and E16 rats showed that all CTGF-stained fibrocytes expressed 4-HNE (white arrows in **M–R**) compared to unexposed rats (white arrows in **K,L**). Dashed square boxes in **(A,C,E,G)** denote the approximate locations of the higher-magnification images shown for each group. Yellow arrows point to 4-HNE-stained fibrocytes that do not express CTGF. Asterisks in **(F,H)** indicate blood vessels. Cell nuclei (blue) were counterstained with DAPI. *p < 0.05; ^***^p < 0.001. Scales bars: 100 μm in **(G)**; 20 μm in **(H,R)**. *n* = 4 for each animal group.

As damage to type IV fibrocytes has been associated to noise-induced trauma (Adams, 2009), the possible contribution of these cells immunostained for CTGF, a type IV fibrocyte marker, to ARHL and NAP was also evaluated. In unexposed (NE6) rats, type IV fibrocytes stained strongly for CTGF (Figures 5A,B). Following long-term noise exposure, both young/adult (E6, Figures 5C,D) and aged (E16, Figures 5G,H) rats displayed a significant decrease in the immunostaining of type IV fibrocytes relative to unexposed NE6 (Figures 5A,B) and NE16 (Figures 5E,F) animals. CTGF-stained fibrocytes were more darkly stained in the NE16 group (Figures 5E,F) than in the E6 and E16 groups, but not the NE6 group (Figure 5). Densitometric analysis of CTGF immunostaining in the spiral ligament using ANOVA also revealed a significant interaction between noise and aging [*F*(4, 24) = 17,25, *p* < 0.001] over the levels of CTGF. The Scheffé *post hoc* test demonstrated a statistically significant decrease in the mean gray value for CTGF in the E6, NE16, and E16 groups (–20.62, –11.16, and –26.18%, respectively) when compared to CTR and NE6 groups (Figures 5I,J).

Next, we performed CTGF/4-HNE double-labeling to evaluate whether CTGC-stained fibrocytes also express 4-HNE, a marker indicative of fibrocyte damage. Compared with NE6 rats (Figure 5K), CTGF-stained type IV fibrocytes in noise-exposed (E6) rats (white arrows in Figure 5M) and aging rats (NE16 and E16 groups) (arrows in Figures 5O,Q, respectively) also expressed 4-HNE, indicating elevated levels of lipid peroxidation product within these cells (Figures 5N,P,R; respectively). Note that all CTGF-stained type IV fibrocytes were co-stained with 4-HNE (white arrows in Figures 5K–R); however, not all 4-HNE-stained fibrocytes expressed CTGF (yellow arrows in Figures 5M–R).

#### Metabolic Oxidative Stress

**FIGURE 6:**
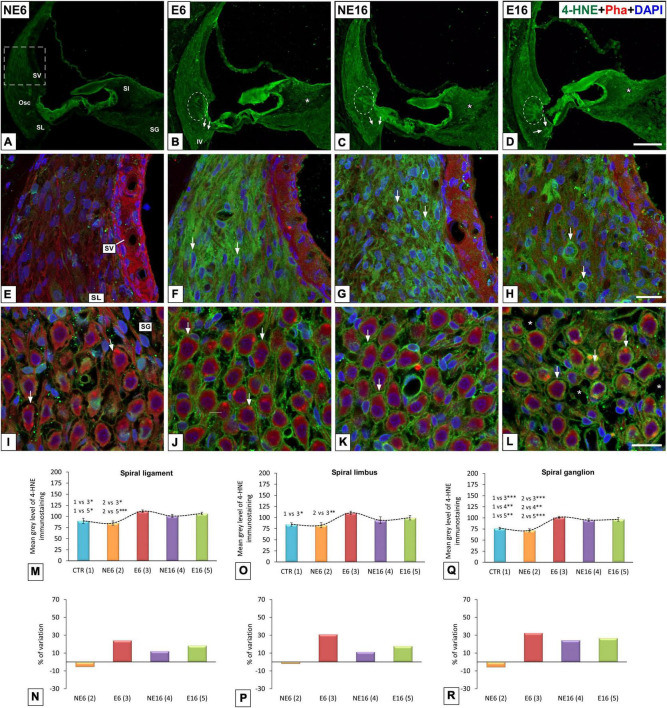
Digital images showing 4-HNE-immunostaining in young/adult and aging cochleae exposed to NAP. **(A–D)** Cochlear midmodiolar sections showing increases in 4-HNE-immunostaining (green) in the spiral ligament, spiral limbus, organ of Corti, and spiral ganglion in E6 **(B,F,J)**, NE16 **(C,G,K),** and E16 **(D,H,L)** rats compared to unexposed **(A,E,I)** control rats. The quantification of immunostaining and percentage of variation for each cochlear structure of interest in each group corroborated these increases **(M–R)**. The dashed square box in **(A)** represents the approximate location of the higher-magnification images shown for each group. Dashed ovals in **(B–D)** encircle outer sulcus root cells. Arrows point to 4-HNE-stained cells, and asterisks in L indicate blood vessel dilation. Phalloidin (red) was used to label staining actin filaments and cell nuclei (blue) were counterstained with DAPI.Osc, outer sulcus; SL, spiral ligament; SG, spiral ganglion; Sl, spiral limbus; SV, stria vascularis. *p < 0.05; ^**^p < 0.01; ^***^p < 0.001. Scales bars: 100 μm in **(D)**; 20 μm in **(H,L)**. *n* = 4 for each animal group.

In addition to increased levels of 4-HNE in type IV fibrocytes and root cells in E6 (arrows and dashed ovals in Figure 6B), NE16 (arrows and dashed ovals in Figure 6C), and E16 (arrows and dashed ovals in Figure 6D) rats, they were also increased in types I, II, III and V fibrocytes in the spiral ligament, organ of Corti, spiral limbus, and spiral ganglion in these same groups (Figures 6B–D,F–H,J–L, respectively) compared with those of unexposed rats (NE6), where the expression of this lipid peroxidation product was very weak (Figures 6A,E,I). Degenerative cellular changes were particularly evident in the oldest age group exposed to noise (E16), in which alterations in fibrocyte morphology (arrows in Figure 6H) and loss of spiral ganglion integrity (arrows in Figure 6L) were more extensive relative to the other groups (NE6, E6, NE16). Notably, 4-HNE-expressing fibrocytes in E16 rats displayed an irregular, rounded morphology with no evident extending processes (arrows in Figure 6H).

ANOVA showed a significant effect of age and noise exposure on the mean gray level for 4-HNE immunostaining in the spiral ligament [*F*(4, 38) = 7.12, *p* < 0.001], spiral limbus [*F*(3, 26) = 5.38, *p* < 0.01], and spiral ganglion [*F*(4, 30) = 19.03, *p* < 0.001]. In addition, further analysis using the Scheffé *post hoc* test indicated that the mean gray values for 4-HNE in the spiral ligament of E6 and E16 rats were significantly increased (+ 24.20% and + 18.40%, respectively) as compared to those in both the CTR and NE6 groups (Figures 6M,N). In the spiral limbus, the mean gray values for 4-HNE were significantly higher in E6 rats (+ 30.97%) compared to those of the CTR (*p* < 0.05) and NE6 (*p* < 0.01) groups, but not to the NE16 and E16 groups (Figures 6O,P). In the spiral ganglion, meanwhile, the mean gray levels were significant higher in the E6, NE16, and E16 groups (+32.61, +24.45%, and +26.82%, respectively) relative to the CTR and the NE6 groups (Figures 6Q,R).

### Changes in Antioxidant Enzymes/Inflammation Mediator Gene Expression Associated With Age-Related Hearing Loss and Noise-Accelerated Presbycusis

**FIGURE 7:**
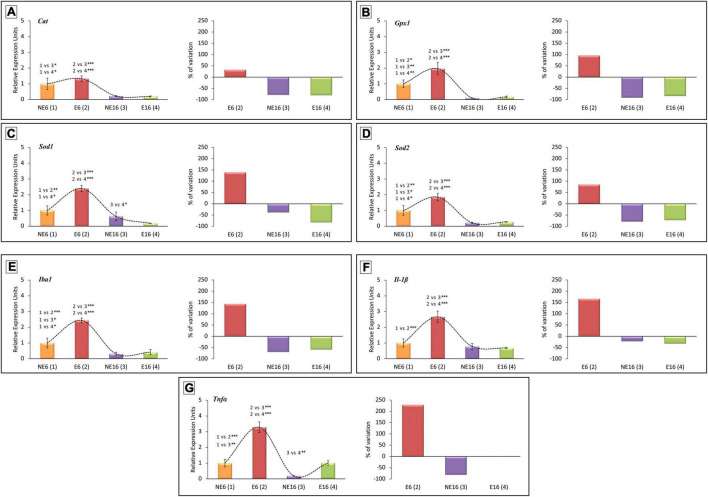
Quantitative PCR expression profiles of genes related to antioxidant capacity **(A–D)** and inflammation **(E–G)** in the cochlea of young/adult and aging rats exposed to NAP. In rats exposed to NAP (E6), the relative expression of *Gpx1* **(B)**, *Sod1* **(C)**, and *Sod2* **(D)** were significantly higher than NE6 baseline levels and NE16 and E16 rats (see section “Results”). However, *Cat* **(A)**, *Gpx1* **(B)**, and *Sod2* **(D)** transcription levels in the cochleae of older unexposed rats (NE16) were significantly lower than in NE6 and E6 rats. Similarly, the expression of all antioxidant genes evaluated in the cochleae of E16 rats decreased relative to the NE and E6 groups. **(A–D)** The expression of the inflammation-related genes *Iba1* **(E)**, *Il-1*β **(F)**, and *TNF*α **(G)** increased significantly in E6 rats relative to unexposed control baseline and aging rats. As aging progressed, expression of *Iba1* **(E)** and *TNF*α **(G)** genes in NE16 and E16 cochleae decreased significantly. *p < 0.05; ^**^p < 0.01; ^***^p < 0.001. Data presented as mean ± SEM of triplicate samples (*n* = 4 for each animal group). *n* = 4 for each animal group.

Lastly, we explored whether noise exposure affected the expression levels of antioxidant enzymes and inflammation mediator genes in the cochlea, and thus contribute to presbycusis and/or NAP. Regarding the antioxidant-related genes, ANOVA indicated a significant interaction between noise and age over the transcript levels of catalase (*Cat*) [*F*(3, 38) = 9.48, *p* < 0.001], glutathione peroxidase 1 (*Gpx1*) [*F*(3, 41) = 15.06, *p* < 0.001], superoxide dismutase 1 (*Sod1*) [*F*(3, 41) = 21.21, *p* < 0.001], and *Sod2* [*F*(3, 41) = 19.29, *p* < 0.001]. As shown in Figure 7, further evaluation using the Scheffé *pos-hoc* test revealed that the mRNA levels of *Gpx1*, *Sod1*, and *Sod2* in E6 rats were significantly increased (+ 96.26%, + 139.40%, and + 85.42%, respectively) compared to those in animals in the NE6, NE16, and E16 groups (Figures 7A–D). The mRNA levels of *Cat*, *Gpx1*, and *Sod2* in the cochleae of NE16 rats were significantly decreased (–78.22, –90.49, and –78.60%, respectively) compared to those of the NE6 and E6 groups (Figures 7A–D). Similarly, there were also significant declines in the mRNA expression levels of *Cat*, *Gpx1*, *Sod1*, and *Sod2* (–81.92, –79.89, –83.01, and –72.76%, respectively) in noise-exposed aged (E16) animals relative to younger unexposed (NE6) and NAP-exposed (E6) rats (Figures 7A–D).

Regarding inflammation mediator genes, ANOVA results demonstrated that there was also a significant interaction between noise and age on the transcript levels of *Iba1* [*F*(3, 41) = 31.17, *p* < 0.001], *Il-1*β [*F*(3, 44) = 13.85, *p* < 0.001] and tumor necrosis factor-alpha (*TNF-*α) [*F*(3, 44) = 35.03, *p* < 0.001]. Scheffé’s *post-hoc* test showed that the transcript levels of *Iba1*, *Il-1*β, and *TNF*α were significantly increased in E6 rats (+ 143.74, + 166.23, and + 228.74%, respectively) compared to levels in NE6, NE16 and E16 animals (Figures 7E–G). In non-exposed aged (NE16) animals, the expression levels of *Iba1* and *TNF*α were significantly lower (–69.26 and –81.03%, respectively) than those of unexposed (NE6) and noise-exposed (E6) adult rats (Figures 7E–G). In exposed aged E16 rats, the mean values for the transcript levels of *Iba1* were significantly reduced (–58.31%) when compared with those of non-exposed (NE6) and noise-exposed (E6) rats (Figures 7E–G). Although there was a significant increase in *TNF*α transcript levels in the E16 group compared with age-matched unexposed rats (NE16), the mean values were similar to those observed in NE6 rats (Figure 7G).

### Cochlear Inflammation

**FIGURE 8:**
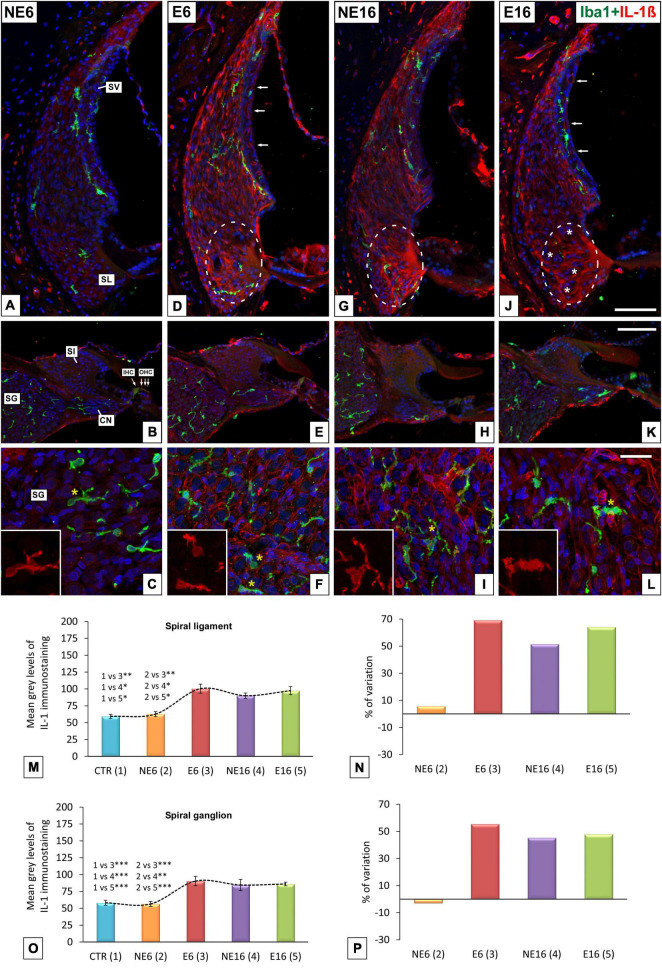
Double immunohistochemical staining for Iba1 (green) and Il-1β (red) in young/adult and aging cochleae exposed to NAP. Relative to unexposed rats **(A)**, the morphology and distribution of Iba1-stained cells in the spiral ligament was similar across animal groups **(D,G,J)**. However, microglia displayed a dystrophic morphology, with fragmented and retracted processes in the spiral ganglia of E6 (* in **E,F**), NE16 (* in **H,I**), and particularly E16 (* in **K,L**) rats relative to unexposed rats **(B,C)**. Elevated levels of Il-1β in type IV fibrocytes, outer sulcus/root cells (dashed ovals in **D,G,J**) and the spiral ganglia **(C,F,H,I,L)** were detected in E6, NE16, and E16 groups, and these increases were confirmed *via* densitometric analysis using ANOVA **(M–P)**. Arrows in **(D,G,J)** indicate increased cytokine expression in blood capillaries in the stria vascularis. White asterisks in **(J)** indicate fibrocyte loss whereas yellow asterisks in **(C,F,I,L)** indicate Iba1-stained cells. Cell nuclei (blue) were counterstained with DAPI.CN, cochlear nerve; IHC, inner hair cell; OHC, outer hair cells; SL, spiral ligament; SG, spiral ganglion; Sl, spiral limbus; SV, stria vascularis. *p < 0.05; ^**^p < 0.01; ^***^p < 0.001. Scales bars: 100 μm in **(J,K)**; 20 μm in **(L)**. *n* = 4 for each animal group.

Next, to further assess whether repeated, long-term exposure to a noise of short-duration triggers cochlear inflammation-related events, alterations in microglial responses, tested by immunocytochemistry with the marker of activated microglia Iba 1, and levels of the proinflammatory cytokine IL-1β were evaluated in the auditory receptor of young/adult and aged rats. In NE6 rats, Iba1-stained microglia were scarce in the spiral ligament (green cells in Figure 8A) and the spiral ganglion (green cells in Figures 8B,C) and showed a bipolar and ramified morphology with long, thin extending processes. No differences were found in microglial distribution and morphology in the spiral ligament between exposed (E6) and unexposed (NE6) rats (compare Figure 8D with Figure 8A). However, noise-induced alterations in microglial phenotype were apparent in the spiral ganglion of E6 rats, where Iba1-stained cells displayed a distinctly heterogenous distribution, with microglia showing shorter, thicker processes (compare Figures 8E,F with Figures 8B,C). With increasing age (NE16 and E16; Figures 8G–L), microglial responses in the spiral ligament remained unchanged, although Iba1-stained cells in the spiral ganglion exhibited a dystrophic morphology, characterized by irregular cell bodies with fragmented and retracted processes (yellow asterisks in Figures 8I–L). The latter results indicate that the combination of age and continuous noise exposure had a more severe impact on microglia in the spiral ganglion (yellow asterisk in Figure 8L).

The levels of IL-1β, a key regulator of inflammation and angiogenesis (Fahey and Doyle, 2019), were also assessed. The expression levels of IL-1β, indicative of cochlear inflammation, were higher in the cochlear lateral wall and spiral ganglion of rats in the E6, NE16, and E16 groups than in those of non-exposed (NE6) animals (Figure 8). Although this pro-inflammatory cytokine was widely distributed throughout the spiral ligament, the highest expression was detected in type IV fibrocytes and outer sulcus cells/root cells area in E6 (dashed oval in Figure 8D), NE16 (dashed oval in Figure 8G), and E16 (dashed oval in Figure 8J) rats. Notably, in the latter group, there was a reduction in the number of IL-1β-stained fibrocytes, possibly due to the combined effect of aging and noise exposure on these cells (asterisks in Figure 8J). Increased IL-1β-expressing cells were also observed in the spiral ganglion of E6 (Figures 8E,F), NE16 (Figures 8H,I), and E16 (Figures 8K,L) rats compared with the non-exposed group (Figures 8B,C). A marked increase in IL-1β expression was seen in neurons and vasculature, which was particularly evident in the oldest animals (NE16 and E16; Figures 8I–L).

ANOVA showed a significant effect of the interaction between age and noise on IL-1β immunostaining levels in both the spiral ligament [*F*(4, 23) = 17.11, *p* < 0.001] and the spiral ganglion [*F*(4, 21) = 8.60, *p* < 0.001]. Scheffé’s *post hoc* test showed that the mean gray values for IL-1β in the spiral ligament in the E6, NE16, and E16 groups were significantly increased (+ 69.16%, + 51.50%, and + 64.22%, respectively) relative to those of both the CTR and NE6 groups (Figures 8M,N). Similarly, in the spiral ganglion, the mean gray values for IL-1β in the E6, NE16, and E16 groups were significantly increased (+ 55.48%, + 43.35%, and + 48.01%, respectively) compared to the CTR and NE6 groups (Figures 8O,P).

## Discussion

The present results demonstrate that exposure to repetitive long-term short-duration noise overstimulation in young Wistar rats, starting at the age of 3 months, aggravates age-dependent hearing deterioration, as seen in significantly higher thresholds and larger threshold shifts in aged, 16-month-old, noise-exposed animals (E16), compared with age-matched, non-exposed animals (NE16). In response to NAP protocol, significant increases in auditory threshold and decreases in waves amplitudes, relative to unexposed 3-month-old, were already observed in E6 rats. In correlation with such threshold shifts, rats in the E6 group exhibited early SV alterations and loss of spiral limbus fibrocytes relative to unexposed rats. On the other hand, NE16 rats showed age-related loss of type IV fibrocytes in the spiral ligament and loss of spiral ganglion cells that, when combined with noise exposure (E16), resulted in increased vulnerability of lateral wall fibrocytes, and also degeneration of SGN. Additionally, the effect of NAP on adult (E6) and aged (E16) animals resulted in increased NKA-immunostaining in the SV, decreased levels of CTGF-stained type IV fibrocytes, increased expression of 4-HNE, and prolonged cochlear inflammatory response.

Although noise is an important risk factor for the onset and/or progression of presbycusis, the exact nature of their interrelation, and its impact on vulnerable cochlear structures and auditory processing are not clearly understood (Bielefeld et al., 2010; Fetoni et al., 2011). Previous research on animal models of NIHL and ARHL have shown that these degenerative diseases are driven by similar pathogenic events involved in decline of cochlear function, including inflammation, increased oxidative stress, and degenerative changes in cochlear vasculature (Fetoni et al., 2011, 2013; Alvarado et al., 2015, 2018, 2020; Tavanai and Mohammadkhani, 2017). Therefore, the coexistence of these common etiopathogenic mechanisms underlying hearing loss would have a significant impact on cochlear tissue damage, inducing premature presbycusis. Physiological studies leading to accelerated long-term decline of auditory function in response to noise exposure have demonstrated that either single or repeated overstimulation with subtraumatic or traumatic sound levels (higher than 85dB SPL) has a deleterious impact on hearing during aging, accelerating and/or aggravating presbycusis (Gates and Mills, 2005; Kujawa and Liberman, 2006; Campo et al., 2011; Fernandez et al., 2015; Alvarado et al., 2019). Consistent with these observations, our findings demonstrate that repeated daily exposure for 1 h to 110 dB SPL noise induces statistically significant increases in auditory threshold and threshold shifts in young adult rats. These include reduction in amplitude and alterations in morphology of ABR waves similar to that observed in presbycusic aged rats. However, it should be highlighted that there is a critical age period (Kujawa and Liberman, 2006; Alvarado et al., 2015), that for Wistar rats is between 3 and 6 months of age, during which the effects of the interaction between noise and age are stronger, and prolonged noise exposure each day for even a short period of time is sufficient to induce NAP.

Studies in animal models of ARHL mimicking aspects of the human clinical condition have shown that this sensory disability, which is characterized by metabolic, sensory, and neural phenotypes, results from pathological changes in the function and structure of vulnerable cochlear regions that collectively impact hearing loss progression (Kalinec et al., 2017; Watson et al., 2017). Previous studies in CBA/J and CBA/CaJ mice have demonstrated that different inbred mouse strains exhibit different aspects of presbycusis. For instance, whereas CBA/J mice show sensorineural presbycusis characterized by delayed high-frequency hearing loss along with loss of outer hair cells and degeneration of the spiral ganglion, CBA/CaJ mice show aspects of strial presbycusis including accelerated hearing loss, strial degeneration, declined endocochlear potential, and loss of outer sulcus/root cells (Sha et al., 2008; Ohlemiller, 2009; Ohlemiller et al., 2010). C57BL/6J (B6) mice show early progressive high-frequency hearing loss and degeneration of the lateral wall, sensory receptors, and SGN (Li and Borg, 1991; Di Girolamo et al., 2001; Ohlemiller, 2009; Carraro and Harrison, 2016). Fisher 344 albino rats show strial pathology and outer hair cell loss correlated with progressive hearing loss by 12 months of age (Buckiova et al., 2007; Bielefeld et al., 2008; Syka, 2010; Balogová et al., 2018). Consistent with these studies, our results demonstrate that aging Wistar rats (NE16) display SV dystrophy, loss of type IV fibrocytes in the lateral wall, and loss of fibrocytes in the spiral limbus, suggesting that fibrocyte degeneration contributes to metabolic/strial presbycusis in this animal model. Similar degenerative cellular patterns in cochlear fibrocytes coupled with hearing dysfunction have been reported in aged 129S6/SvEv and C57BL/6 mice (Zheng et al., 1999; Yoshida et al., 2000; Hequembourg and Liberman, 2001; Ohlemiller and Gagnon, 2004). Because the accelerated presbycusic 6-month-old rats presented physiological characteristics similar to those observed in unexposed old rats (NE16), it would be logical to expect that they also present early pathogenic features like those observed in the unexposed group. Consistent with previous studies in noise-exposed mice (Wang et al., 2002; Shin et al., 2019), our data confirm that E6 rats shared the alterations observed in older unexposed animals, including initial strial pathology and fibrocytes loss in the spiral limbus. Our results also show that when ARHL and NIHL coexist, as in E16 animals, further structural damage occurs including loss of type IV fibrocytes and SGN, suggesting accelerated onset and/or progression of hearing loss.

Aging and noise-exposure also impacted CTGF levels. In this regard, unexposed aged rats (NE16) and NAP-exposed rats (E6 and E16) showed decreased CTGF staining and increased cytotoxic levels of 4-HNE in type IV fibrocytes, relative to the NE6 group. These results are supported by previous studies showing that CTGF-stained type IV fibrocytes in CBA/CaJ mice are extremely vulnerable to noise trauma (6 h post-exposure at 8–16 kHz and to 95 dB) (Wang et al., 2002; Adams, 2009), implying that deteriorated and dysfunctional fibrocytes contribute to gradual hearing loss. However, despite that the progression of age-related functional disability differs both across and within species, the prevailing view is that metabolic presbycusis is predominant in ARHL, with the consequent alteration of endocochlear potential indispensable for proper auditory receptor function (Fetoni et al., 2011; Keithley, 2019; Ohlemiller, 2019).

The ubiquitous enzyme complex NKA, located in cell plasma membranes in the SV and the spiral ligament, is critical for maintenance of the electrochemical composition of the endolymph in the inner ear (Schulte and Adams, 1989; Cate et al., 1994; Nakazawa et al., 1995; Erichsen, 1996; Gratton et al., 1996; Delprat et al., 2007; Patuzzi, 2011; Liu et al., 2017). Accumulating evidence suggests that dysfunction of lateral wall structures (spiral ligament and SV) leading to disruption of the endocochlear potential affects hearing ability. Aged gerbils, mice, and rats display atrophy of the cochlear lateral wall, which correlates with decreased NKA activity and immunostaining (Schulte and Schmiedt, 1992; Gratton et al., 1995, 1997; Spicer and Schulte, 2002; Buckiova et al., 2007; Ding et al., 2018). Our findings in 16-month-old unexposed cochleae show age-related decreases in immunostaining of NKA-expressing type II fibrocytes, which are essential for regulating potassium homeostasis. This provides evidence of lateral wall pathology and ATPase-dysregulation, leading to declined endocochlear potential and contributing to ARHL. In the present study, adult (E6) and aged (E16) rats exposed to NAP showed decreased NKA-immunostaining in the spiral ligament and the spiral ganglion relative to unexposed rats, however, staining in the SV was significantly increased relative to that in the NE6 group. Noise-exposure associated perturbations in NKA levels in the cochlear spiral ligament have been described previously (Hsu et al., 2000; Yamaguchi et al., 2014). In mice, *in vivo* acoustic noise stimulation (1 h–8 kHz octave band noise at 110 dB SPL) results in decreased mRNA levels of Na +, K + -ATPase in spiral ligament fibrocytes 2 h post-exposure which remains until day seven (Yamaguchi et al., 2014). However, white noise exposure (10 min or 40 h at 105 dB) in adult guinea pigs leads to a significant decline in NKA activity 7 days post-exposure in lateral wall structures and followed by a return to basal levels on days 10 and 28 (Hsu et al., 2000, 2002). In these studies, Na +, K + -ATPase activity was evaluated using different noise parameters (duration, intensity, and noise paradigm) and at shorter survival times. The noise-induced increases in NKA in the SV of E6 and E16 cochleae observed in the present study may reflect an ion homeostatic response to chronic daily and short-duration noise stimulation as an attempt to preserving cochlear function.

Another cellular hallmark in the pathogenesis of ARHL and NIHL is the imbalance of redox homeostasis in the injured cochlea. Studies in chinchillas, mice, and rats have shown that aging and noise trauma *in vivo* increase oxidative stress biomarkers such as 4-HNE in lateral cochlear wall structures, organs of Corti, and spiral ganglion, and this correlates with hearing loss (Henderson et al., 2006; Jiang et al., 2007; Du et al., 2011; Yamaguchi et al., 2014; Fetoni et al., 2015a,^2019^; Wang et al., 2019). Increased antioxidant defenses to minimize oxidative stress damage have been reported in the injured cochlea (Fetoni et al., 2019). Upregulation of *Cat* expression by 10 days and *Gpx1* expression between 1 and 10 days after exposure to broadband noise (118 dB SPL for 4 h a day for 4 consecutive days) suggests that *Gpx1* plays a key role in early cochlear enzyme-mediated antioxidation responses to NIHL (Alvarado et al., 2020). In addition, our present findings show that NAP significantly increases *Cat*, *Gpx1*, *Sod1*, and *Sod2* in E6 rats relative to unexposed rats (NE6) and unexposed and noise-exposed aging animals (NE16 and E16), suggesting that early noise exposure increases cochlear susceptibility to aging. We also report that the transcription levels of all four antioxidant enzymes evaluated decreased significantly in both unexposed (NE16) and exposed (E16) aged animals, suggesting that antioxidant capacity is depleted in these animals. Accumulation of free radical damage and decline in the expression of major antioxidant enzymes in cochlear tissue accelerates age-related oxidative damage (Staecker and Zheng, 2001; Jiang et al., 2007; Menardo et al., 2012).

When cultures from murine SL fibrocytes are stimulated by proinflammatory cytokines IL-1β or TNFα, they secrete cellular mediators involved in prolonging inflammation (Yoshida et al., 1999; Ichimiya et al., 2000). Given that these cells may regulate inflammatory-associated events in response to cochlear aging or noise injury, dysfunctional fibrocytes may have a profound impact on hearing function (Fujioka et al., 2014; Okano, 2014; Watson et al., 2017; Peeleman et al., 2020). In the present study, we further demonstrate chronic inflammation characterized by increased *IL-1*β protein levels primarily in type IV fibrocytes in the spiral ligaments and spiral ganglia of adult NAP-exposed, aged unexposed, and aged NAP-exposed rats. However, our results provide evidence of increased *IL-1*β and *TNF*α transcription in noise-exposed cochleae (E6) but not in aged animals (NE16 and E16). Previous studies have also shown noise-induced upregulation of IL-1β and TNFα in cochlear fibrocytes and reactive microglia (Fujioka et al., 2006; Tornabene et al., 2006; Fuentes-Santamaría et al., 2017). Decreased IL-1β and TNFα expression has also been reported in the perilymph of 2-year-old mice relative to younger mice, suggesting that aging and dysfunctional cochlear fibrocytes are not capable of cytokine biosynthesis and/or secretion (Landegger et al., 2019). Our results also showed that macrophages in the spiral ligament were unchanged in E6, NE16, and E16 groups, suggesting that their contribution to the inflamed spiral ligament is primarily regulated by fibrocytes. Macrophages in the spiral ganglia in the same groups switched to an active phenotype, suggesting that microglia also actively contribute to aging and/or noise-induced cochlear inflammation.

In summary, the present findings support the notion that a common pathogenic pathway is involved in NIHL and ARHL genesis. They demonstrate that noise and aging target similar cochlear cell types through similar etiopathogenic mechanisms including inflammation and oxidative stress, and this results in early presbycusis. This implies that the comorbidity between noise and aging, acting in a synergistic and/or redundant manner, leads to premature long-term progressive deterioration of auditory function that accelerates/aggravates presbycusis.

## Data Availability Statement

The original contributions presented in the study are included in the article/supplementary material, further inquiries can be directed to the corresponding author/s.

## Ethics Statement

All procedures involving the use and care of the animals were approved by the Ethics Committee on Animal Experimentation at the University of Castilla-La Mancha (Permit Number: PR-2019-02-05) and conformed to European Union (Directive 2010/63/EU) and Spanish (R.D. 53/2013; Law 32/2007) regulations for the care and use of animals in research.

## Author Contributions

VF-S and JA: study concept, design, and drafting of the manuscript. VF-S, JA, SM, PM-R, MG-U, and JC-S: acquisition of data. JA, VF-S, and SM: statistical analysis and interpretation of data. JA, VF-S, PM-R, SM, and JJ: critical revision of the manuscript for important intellectual content. JJ, VF-S, and JA: obtaining funding. All authors have read and agreed to the published version of the manuscript.

## Conflict of Interest

The authors declare that the research was conducted in the absence of any commercial or financial relationships that could be construed as a potential conflict of interest.

## Publisher’s Note

All claims expressed in this article are solely those of the authors and do not necessarily represent those of their affiliated organizations, or those of the publisher, the editors and the reviewers. Any product that may be evaluated in this article, or claim that may be made by its manufacturer, is not guaranteed or endorsed by the publisher.

## References

[B1] Adams J. C. (2009). Immunocytochemical traits of type iv fibrocytes and their possible relations to cochlear function and pathology. *J. Assoc. Res. Otolaryngol.* 10 369–382. 10.1007/s10162-009-0165-z 19277783 PMC3084383

[B2] Alvarado J. C. Fuentes-Santamaría V. Gabaldón-Ull M. C. Blanco J. L. Juiz J. M. (2014). Wistar rats: a forgotten model of age-related hearing loss. *Front. Aging Neurosci.* 6:29. 10.3389/fnagi.2014.00029 24634657 PMC3942650

[B3] Alvarado J. C. Fuentes-Santamaría V. Gabaldón-Ull M. C. Jareño-Flores T. Miller J. M. Juiz J. M. (2016). Noise-induced “toughening” effect in wistar rats: enhanced auditory brainstem responses are related to calretinin and nitric oxide synthase upregulation. *Front. Neuroanat.* 10:19. 10.3389/fnana.2016.00019 27065815 PMC4815363

[B4] Alvarado J. C. Fuentes-Santamaría V. Gabaldón-Ull M. C. Juiz J. M. (2018). An oral combination of vitamins a, c, e, and Mg++ improves auditory thresholds in age-related hearing loss. *Front. Neurosci.* 12:527. 10.3389/fnins.2018.00527 30108480 PMC6079267

[B5] Alvarado J. C. Fuentes-Santamaría V. Gabaldón-Ull M. C. Juiz J. M. (2019). Age-related hearing loss is accelerated by repeated short-duration loud sound stimulation. *Front. Neurosci.* 13:77. 10.3389/fnins.2019.00077 30872984 PMC6402475

[B6] Alvarado J. C. Fuentes-Santamaría V. Henkel C. K. (2009). Rapid modifications in calretinin immunostaining in the deep layers of the superior colliculus after unilateral cochlear ablation. *Hear. Res.* 247 78–86. 10.1016/j.heares.2008.10.005 19017539

[B7] Alvarado J. C. Fuentes-Santamaría V. Henkel C. K. Brunso-Bechtold J. K. (2004). Alterations in calretinin immunostaining in the ferret superior olivary complex after cochlear ablation. *J. Comp. Neurol.* 470 63–79. 10.1002/cne.11038 14755526

[B8] Alvarado J. C. Fuentes-Santamaría V. Jareño-Flores T. Blanco J. L. Juiz J. M. (2012). Normal variations in the morphology of auditory brainstem response (ABR) waveforms: a study in wistar rats. *Neurosci. Res.* 73 302–311. 10.1016/j.neures.2012.05.001 22595234

[B9] Alvarado J. C. Fuentes-Santamaría V. Melgar-Rojas P. Gabaldón-Ull M. C. Cabanes-Sanchis J. J. Juiz J. M. (2020). Oral antioxidant vitamins and magnesium limit noise-induced hearing loss by promoting sensory hair cell survival: role of antioxidant enzymes and apoptosis genes. *Antioxidants* 9:1177. 10.3390/antiox9121177 33255728 PMC7761130

[B10] Alvarado J. C. Fuentes-Santamaría V. Melgar-Rojas P. Valero M. L. Gabaldón-Ull M. C. Miller J. M. (2015). Synergistic effects of free radical scavengers and cochlear vasodilators: a new otoprotective strategy for age-related hearing loss. *Front. Aging Neurosci.* 7:86. 10.3389/fnagi.2015.00086 26029103 PMC4432684

[B11] Balogová Z. Popelář J. Chiumenti F. Chumak T. Burianová J. S. Rybalko N. (2018). Age-related differences in hearing function and cochlear morphology between male and female fischer 344 rats. *Front. Aging Neurosci.* 9:28. 10.3389/fnagi.2017.00428 29354051 PMC5758597

[B12] Bermúdez-Muñoz J. M. Celaya A. M. Hijazo-Pechero S. Wang J. Serrano M. Varela-Nieto I. (2020). G6PD overexpression protects from oxidative stress and age-related hearing loss. *Aging Cell* 19:e13275. 10.1111/acel.13275 33222382 PMC7744953

[B13] Bielefeld E. C. Coling D. Chen G.-D. Li M. Tanaka C. Hu B.-H. (2008). Age-related hearing loss in the fischer 344/NHsd rat substrain. *Hear. Res.* 241 26–33. 10.1016/j.heares.2008.04.006 18508213 PMC2556048

[B14] Bielefeld E. C. Tanaka C. Chen G. Henderson D. (2010). Age-related hearing loss: is it a preventable condition? *Hear. Res.* 264 98–107. 10.1016/j.heares.2009.09.001 19735708 PMC2868117

[B15] Bowl M. R. Dawson S. J. (2014). The mouse as a model for age-related hearing loss - a mini-review. *Gerontology* 61 149–157. 10.1159/000368399 25471225

[B16] Buckiova D. Popelar J. Syka J. (2007). Aging cochleas in the F344 rat: morphological and functional changes. *Exp. Gerontol.* 42 629–638. 10.1016/j.exger.2007.02.007 17442517

[B17] Bustin S. A. Benes V. Garson J. A. Hellemans J. Huggett J. Kubista M. (2009). The miqe guidelines: minimum information for publication of quantitative real-time PCR experiments. *Clin. Chem.* 55 611–622. 10.1373/clinchem.2008.112797 19246619

[B18] Campo P. Venet T. Rumeau C. Thomas A. Rieger B. Cour C. (2011). Impact of noise or styrene exposure on the kinetics of presbycusis. *Hear. Res.* 280 122–132. 10.1016/j.heares.2011.04.016 21616132

[B19] Carraro M. Harrison R. V. (2016). Degeneration of stria vascularis in age-related hearing loss; a corrosion cast study in a mouse model. *Acta Otolaryngol.* 136 385–390. 10.3109/00016489.2015.1123291 26824717

[B20] Carroll Y. I. Eichwald J. Scinicariello F. Hoffman H. J. Deitchman S. Radke M. S. (2017). Vital signs: noise-induced hearing loss among adults — United States 2011–2012. *MMWR Morb. Mortal. Wkly. Rep.* 66 139–144. 10.15585/mmwr.mm6605e3 28182600 PMC5657963

[B21] Cate W.-J. F. ten Curtis L. M. Rarey K. E. (1994). Na,K-ATPase α and β subunit isoform distribution in the rat cochlear and vestibular tissues. *Hear. Res.* 75 151–160. 10.1016/0378-5955(94)90066-3 8071142

[B22] Chen Z. Zhang Y. Zhang J. Zhou R. Zhong Z. Wei C. (2021). Cochlear Synaptopathy: a primary factor affecting speech recognition performance in presbycusis. *BioMed. Res. Int.* 2021 1–7. 10.1155/2021/6667531 34409106 PMC8367534

[B23] Church M. W. Adams B. R. Anumba J. I. Jackson D. A. Kruger M. L. Jen K.-L. C. (2012a). Repeated antenatal corticosteroid treatments adversely affect neural transmission time and auditory thresholds in laboratory rats. *Neurotoxicol. Teratol.* 34 196–205. 10.1016/j.ntt.2011.09.004 21963399 PMC3268869

[B24] Church M. W. Hotra J. W. Holmes P. A. Anumba J. I. Jackson D. A. Adams B. R. (2012b). Auditory brainstem response (ABR) abnormalities across the life span of rats prenatally exposed to alcohol. *Alcohol. Clin. Exp. Res.* 36 83–96. 10.1111/j.1530-0277.2011.01594.x 21815896 PMC3210930

[B25] Church M. W. Jen K.-L. C. Anumba J. I. Jackson D. A. Adams B. R. Hotra J. W. (2010). Excess omega-3 fatty acid consumption by mothers during pregnancy and lactation caused shorter life span and abnormal ABRs in old adult offspring. *Neurotoxicol. Teratol.* 32 171–181. 10.1016/j.ntt.2009.09.006 19818397 PMC2839050

[B26] Delprat B. Puel J.-L. Geering K. (2007). Dynamic expression of FXYD6 in the inner ear suggests a role of the protein in endolymph homeostasis and neuronal activity. *Dev. Dyn.* 236 2534–2540. 10.1002/dvdy.21269 17676640

[B27] Di Girolamo S. Quaranta N. Picciotti P. Torsello A. Wolf F. (2001). Age-related histopathological changes of the stria vascularis: an experimental model. *Audiol. Off. Organ Int. Soc. Audiol.* 40 322–326. 10.3109/00206090109073129 11781045

[B28] Ding B. Walton J. P. Zhu X. Frisina R. D. (2018). Age-related changes in Na. K-ATPase expression, subunit isoform selection and assembly in the stria vascularis lateral wall of mouse cochlea. *Hear. Res.* 367 59–73. 10.1016/j.heares.2018.07.006 30029086 PMC7012304

[B29] Du X. Choi C.-H. Chen K. Cheng W. Floyd R. A. Kopke R. D. (2011). Reduced formation of oxidative stress biomarkers and migration of mononuclear phagocytes in the cochleae of chinchilla after antioxidant treatment in acute acoustic trauma. *Int. J. Otolaryngol.* 2011 1–13. 10.1155/2011/612690 21961007 PMC3179894

[B30] Engle J. R. Tinling S. Recanzone G. H. (2013). Age-related hearing loss in rhesus monkeys is correlated with cochlear histopathologies. *PLoS One* 8:e55092. 10.1371/journal.pone.0055092 23390514 PMC3563598

[B31] Erichsen S. (1996). Na,K-ATPase α- and β-isoforms in the developing cochlea of the mouse. *Hear Res.* 100 143–149. 10.1016/0378-5955(96)00105-0 8922988

[B32] Escabi C. D. Frye M. D. Trevino M. Lobarinas E. (2019). The rat animal model for noise-induced hearing loss. *J. Acoust. Soc. Am.* 146 3692–3709. 10.1121/1.5132553 31795685 PMC7480078

[B33] Fahey E. Doyle S. L. (2019). IL-1 family cytokine regulation of vascular permeability and angiogenesis. *Front. Immunol.* 10:1426. 10.3389/fimmu.2019.01426 31293586 PMC6603210

[B34] Fernandez K. A. Jeffers P. W. C. Lall K. Liberman M. C. Kujawa S. G. (2015). Aging after noise exposure: acceleration of cochlear synaptopathy in “recovered”. *Ears. J. Neurosci.* 35 7509–7520. 10.1523/JNEUROSCI.5138-14.2015 25972177 PMC4429155

[B35] Fetoni A. R. De Bartolo P. Eramo S. L. M. Rolesi R. Paciello F. Bergamini C. (2013). Noise-induced hearing loss (NIHL) as a target of oxidative stress-mediated damage: cochlear and cortical responses after an increase in antioxidant defense. *J. Neurosci.* 33 4011–4023. 10.1523/JNEUROSCI.2282-12.2013 23447610 PMC6619303

[B36] Fetoni A. R. Paciello F. Rolesi R. Eramo S. L. M. Mancuso C. Troiani D. (2015a). Rosmarinic acid up-regulates the noise-activated Nrf2/HO-1 pathway and protects against noise-induced injury in rat cochlea. *Free Radic. Biol. Med.* 85 269–281. 10.1016/j.freeradbiomed.2015.04.021 25936352

[B37] Fetoni A. R. Paciello F. Rolesi R. Paludetti G. Troiani D. (2019). Targeting dysregulation of redox homeostasis in noise-induced hearing loss: oxidative stress and ROS signaling. *Free Radic. Biol. Med.* 135 46–59. 10.1016/j.freeradbiomed.2019.02.022 30802489

[B38] Fetoni A. R. Picciotti P. M. Paludetti G. Troiani D. (2011). Pathogenesis of presbycusis in animal models: a review. *Exp. Gerontol.* 46 413–425. 10.1016/j.exger.2010.12.003 21211561

[B39] Fetoni A. R. Troiani D. Petrosini L. Paludetti G. (2015b). Cochlear injury and adaptive plasticity of the auditory cortex. *Front. Aging Neurosci.* 7:8. 10.3389/fnagi.2015.00008 25698966 PMC4318425

[B40] Fischer N. Johnson Chacko L. Glueckert R. Schrott-Fischer A. (2020). Age-dependent changes in the cochlea. *Gerontology* 66 33–39. 10.1159/000499582 31117093

[B41] Fuentes-Santamaría V. Alvarado J. C. Brunso-Bechtold J. K. Henkel C. K. (2003). Upregulation of calretinin immunostaining in the ferret inferior colliculus after cochlear ablation. *J. Comp. Neurol.* 460 585–596. 10.1002/cne.10676 12717716

[B42] Fuentes-Santamaría V. Alvarado J. C. Gabaldón-Ull M. C. Juiz J. M. (2013). Upregulation of insulin-like growth factor and interleukin 1β occurs in neurons but not in glial cells in the cochlear nucleus following cochlear ablation: upregulation of IGF-1 and IL-1β in cochlear nucleus. *J. Comp. Neurol.* 521 3478–3499. 10.1002/cne.23362 23681983

[B43] Fuentes-Santamaría V. Alvarado J. C. Henkel C. K. Brunso-Bechtold J. K. (2007a). Cochlear ablation in adult ferrets results in changes in insulin-like growth factor-1 and synaptophysin immunostaining in the cochlear nucleus. *Neuroscience* 148 1033–1047. 10.1016/j.neuroscience.2007.07.026 17764853

[B44] Fuentes-Santamaría V. Alvarado J. C. Herranz A. S. García-Atarés N. López D. E. (2007b). Morphologic and neurochemical alterations in the superior colliculus of the genetically epilepsy-prone hamster (GPG/Vall). *Epilepsy Res.* 75 206–219. 10.1016/j.eplepsyres.2007.06.005 17628427

[B45] Fuentes-Santamaría V. Alvarado J. C. Juiz J. M. (2012). Long-term interaction between microglial cells and cochlear nucleus neurons after bilateral cochlear ablation. *J. Comp. Neurol.* 520 2974–2990. 10.1002/cne.23088 22351306

[B46] Fuentes-Santamaría V. Alvarado J. C. López-Muñoz D. F. Melgar-Rojas P. Gabaldón-Ull M. C. Juiz J. M. (2014). Glia-related mechanisms in the anteroventral cochlear nucleus of the adult rat in response to unilateral conductive hearing loss. *Front. Neurosci.* 8:319. 10.3389/fnins.2014.00319 25352772 PMC4195288

[B47] Fuentes-Santamaría V. Alvarado J. C. Melgar-Rojas P. Gabaldón-Ull M. C. Miller J. M. Juiz J. M. (2017). The role of glia in the peripheral and central auditory system following noise overexposure: contribution of TNF-α and IL-1β to the pathogenesis of hearing loss. *Front. Neuroanat.* 11:9. 10.3389/fnana.2017.00009 28280462 PMC5322242

[B48] Fuentes-Santamaría V. Alvarado J. C. Rodríguez-de la Rosa L. Juiz J. M. Varela-Nieto I. (2019). Neuroglial involvement in abnormal glutamate transport in the cochlear nuclei of the Igf1-/- mouse. *Front. Cell. Neurosci.* 13:67. 10.3389/fncel.2019.00067 30881288 PMC6405628

[B49] Fuentes-Santamaría V. Alvarado J. C. Taylor A. R. Brunso-Bechtold J. K. Henkel C. K. (2005). Quantitative changes in calretinin immunostaining in the cochlear nuclei after unilateral cochlear removal in young ferrets. *J. Comp. Neurol.* 483 458–475. 10.1002/cne.20437 15700274 PMC1913210

[B50] Fuentes-Santamaria V. Stein B. E. McHaffie J. G. (2006). Neurofilament proteins are preferentially expressed in descending output neurons of the cat the superior colliculus: a study using SMI-32. *Neuroscience* 138 55–68. 10.1016/j.neuroscience.2005.11.045 16426768

[B51] Fujioka M. Kanzaki S. Okano H. J. Masuda M. Ogawa K. Okano H. (2006). Proinflammatory cytokines expression in noise-induced damaged cochlea. *J. Neurosci. Res.* 83 575–583. 10.1002/jnr.20764 16429448

[B52] Fujioka M. Okano H. Ogawa K. (2014). Inflammatory and immune responses in the cochlea: potential therapeutic targets for sensorineural hearing loss. *Front. Pharmacol.* 5:287. 10.3389/fphar.2014.00287 25566079 PMC4274906

[B53] Gates G. A. Mills J. H. (2005). Presbycusis. *The Lancet* 366 1111–1120. 10.1016/S0140-6736(05)67423-5 16182900

[B54] Gordon-Salant S. (2005). Hearing loss and aging: new research findings and clinical implications. *J. Rehabil. Res. Dev.* 42:9. 10.1682/JRRD.2005.01.0006 16470462

[B55] Gourévitch B. Doisy T. Avillac M. Edeline J.-M. (2009). Follow-up of latency and threshold shifts of auditory brainstem responses after single and interrupted acoustic trauma in guinea pig. *Brain Res.* 1304 66–79. 10.1016/j.brainres.2009.09.041 19766602

[B56] Gratton M. A. Schmiedt R. A. Schulte B. A. (1996). Age-related decreases in endocochlear potential are associated with vascular abnormalities in the stria vascularis. *Hear. Res.* 102 181–190. 10.1016/S0378-5955(96)90017-9 8951461

[B57] Gratton M. A. Schulte B. A. Smythe N. M. (1997). Quantification of the stria vascularis and strial capillary areas in quiet-reared young and aged gerbils. *Hear. Res.* 114 1–9. 10.1016/S0378-5955(97)00025-7 9447913

[B58] Gratton M. A. Smyth B. J. Schulte B. A. Vincent D. A. (1995). Na,K-ATPase activity decreases in the cochlear lateral wall of quiet-aged gerbils. *Hear. Res.* 83 43–50. 10.1016/0378-5955(94)00188-V 7607990

[B59] Hao X. Xing Y. Moore M. W. Zhang J. Han D. Schulte B. A. (2014). Sox10 expressing cells in the lateral wall of the aged mouse and human cochlea. *PLoS One* 9:e97389. 10.1371/journal.pone.0097389 24887110 PMC4041576

[B60] He W. Yu J. Sun Y. Kong W. (2020). Macrophages in noise-exposed cochlea: changes. regulation and the potential role. *Aging Dis.* 11 191. 10.14336/AD.2019.0723 32010492 PMC6961779

[B61] Henderson D. Bielefeld E. C. Harris K. C. Hu B. H. (2006). The role of oxidative stress in noise-induced hearing loss. *Ear Hear.* 27 1–19. 10.1097/01.aud.0000191942.36672.f3 16446561

[B62] Herborn C. U. Waldschuetz R. Lauenstein T. C. Goyen M. Lauffer R. B. Moeroey T. (2002). Contrast-enhanced magnetica resonance imaging (MS-325) in a murine model of systemic lupus erythematosus. *Invest. Radiol.* 37 464–469. 10.1097/00004424-200208000-00008 12138363

[B63] Hequembourg S. Liberman M. C. (2001). Spiral ligament pathology: a major aspect of age-related cochlear degeneration in C57BL/6 mice. *J. Assoc. Res. Otolaryngol.* 2 118–129. 10.1007/s101620010075 11550522 PMC3201181

[B64] Hirose K. Discolo C. M. Keasler J. R. Ransohoff R. (2005). Mononuclear phagocytes migrate into the murine cochlea after acoustic trauma. *J. Comp. Neurol.* 489 180–194. 10.1002/cne.20619 15983998

[B65] Hsu C.-J. Chen Y.-S. Shau W.-Y. Yeh T.-H. Lee S.-Y. Lin-Shiau S. Y. (2002). Impact of activities of Na(+)-K(+)-ATPase and Ca2(+)-ATPase in the cochlear lateral wall on recovery from noise-induced temporary threshold shift. *Ann. Otol. Rhinol. Laryngol.* 111 842–849. 10.1177/000348940211100915 12296342

[B66] Hsu C.-J. Shau W.-Y. Chen Y.-S. Liu T.-C. Lin-Shiau S. Y. (2000). Activities of Na+,K+-ATPase and Ca2+-ATPase in cochlear lateral wall after acoustic trauma. *Hear. Res.* 142 203–211. 10.1016/S0378-5955(00)00020-4 10748339

[B67] Hu B. H. Zhang C. Frye M. D. (2018). Immune cells and non-immune cells with immune function in mammalian cochleae. *Hear. Res.* 362 14–24. 10.1016/j.heares.2017.12.009 29310977 PMC5911222

[B68] Ichimiya I. Yoshida K. Hirano T. Suzuki M. Mogi G. (2000). Significance of spiral ligament fibrocytes with cochlear inflammation. *Int. J. Pediatr. Otorhinolaryngol.* 56 45–51. 10.1016/s0165-5876(00)00408-0 11074115

[B69] Jamesdaniel S. Ding D. Kermany M. H. Davidson B. A. Knight P. R. Salvi R. (2008). Proteomic analysis of the balance between survival and cell death responses in cisplatin-mediated ototoxicity. *J. Proteome Res.* 7 3516–3524. 10.1021/pr8002479 18578524 PMC2570323

[B70] Jayakody D. M. P. Friedland P. L. Martins R. N. Sohrabi H. R. (2018). Impact of aging on the auditory system and related cognitive functions: a narrative review. *Front. Neurosci.* 12:125. 10.3389/fnins.2018.00125 29556173 PMC5844959

[B71] Jiang H. Talaska A. E. Schacht J. Sha S.-H. (2007). Oxidative imbalance in the aging inner ear. *Neurobiol. Aging* 28 1605–1612. 10.1016/j.neurobiolaging.2006.06.025 16920227 PMC2453750

[B72] Kalinec G. M. Lomberk G. Urrutia R. A. Kalinec F. (2017). Resolution of cochlear inflammation: novel target for preventing or ameliorating drug-, noise- and age-related hearing loss. *Front. Cell. Neurosci.* 11:192. 10.3389/fncel.2017.00192 28736517 PMC5500902

[B73] Keithley E. M. (2019). Pathology and mechanisms of cochlear aging. *J. Neurosci. Res.* 98 1674–1684. 10.1002/jnr.24439 31066107 PMC7496655

[B74] Kidd A. R. Bao J. (2012). Recent advances in the study of age-related hearing loss: a mini-review. *Gerontology* 58 490–496. 10.1159/000338588 22710288 PMC3766364

[B75] Kujawa S. G. Liberman M. C. (2006). Acceleration of age-related hearing loss by early noise exposure: evidence of a misspent youth. *J. Neurosci.* 26 2115–2123. 10.1523/JNEUROSCI.4985-05.2006 16481444 PMC1855187

[B76] Landegger L. D. Vasilijic S. Fujita T. Soares V. Y. Seist R. Xu L. (2019). Cytokine levels in inner ear fluid of young and aged mice as molecular biomarkers of noise-induced hearing loss. *Front. Neurol.* 10:977. 10.3389/fneur.2019.00977 31632328 PMC6749100

[B77] Li H.-S. Borg E. (1991). Age-related loss of auditory sensitivity in two mouse genotypes. *Acta Otolaryngol.* 111 827–834. 10.3109/00016489109138418 1759567

[B78] Li H.-S. Borg E. (1993). Auditory degeneration after acoustic trauma in two genotypes of mice. *Hear. Res.* 68 19–27. 10.1016/0378-5955(93)90060-E 8376211

[B79] Lin L. H. Talman W. T. (2000). N-methyl-D-aspartate receptors on neurons that synthesize nitric oxide in rat nucleus tractus solitarii. *Neuroscience* 100 581–588. 10.1016/s0306-4522(00)00314-6 11098121

[B80] Liu W. Li H. Edin F. Brännström J. Glueckert R. Schrott-Fischer A. (2017). Molecular composition and distribution of gap junctions in the sensory epithelium of the human cochlea—a super-resolution structured illumination microscopy (SR-SIM) study. *Ups. J. Med. Sci.* 122 160–170. 10.1080/03009734.2017.1322645 28513246 PMC5649321

[B81] Lyu A.-R. Kim T. H. Park S. J. Shin S.-A. Jeong S.-H. Yu Y. (2020). Mitochondrial damage and necroptosis in aging cochlea. *Int. J. Mol. Sci.* 21:2505. 10.3390/ijms21072505 32260310 PMC7177801

[B82] McLean W. J. Smith K. A. Glowatzki E. Pyott S. J. (2009). Distribution of the Na,K-ATPase α subunit in the rat spiral ganglion and organ of corti. *J. Assoc. Res. Otolaryngol.* 10 37–49. 10.1007/s10162-008-0152-9 19082858 PMC2644389

[B83] Melgar-Rojas P. Alvarado J. C. Fuentes-Santamaría V. Gabaldón-Ull M. C. Juiz J. M. (2015). Validation of reference genes for RT–qPCR analysis in noise–induced hearing loss: a study in wistar rat. *PLoS One* 10:e0138027. 10.1371/journal.pone.0138027 26366995 PMC4569353

[B84] Menardo J. Tang Y. Ladrech S. Lenoir M. Casas F. Michel C. (2012). Oxidative stress, inflammation, and autophagic stress as the key mechanisms of premature age-related hearing loss in SAMP8 mouse cochlea. *Antioxid. Redox Signal.* 16 263–274. 10.1089/ars.2011.4037 21923553

[B85] Nagashima R. Yamaguchi T. Tanaka H. Ogita K. (2010). Mechanism underlying the protective effect of tempol and Nω-Nitro-L-arginine methyl ester on acoustic injury: possible involvement of c-jun n-terminal kinase pathway and connexin26 in the cochlear spiral ligament. *J. Pharmacol. Sci.* 114 50–62. 10.1254/jphs.10113FP 20703012

[B86] Nakazawa K. Spicer S. S. Schulte B. A. (1995). Ultrastructural localization of Na,K-ATPase in the gerbil cochlea. *J. Histochem. Cytochem.* 43 981–991. 10.1177/43.10.7560888 7560888

[B87] Nelson E. G. Hinojosa R. (2006). Presbycusis: a human temporal bone study of individuals with downward sloping audiometric patterns of hearing loss and review of the literature. *Laryngoscope* 116 1–12. 10.1097/01.mlg.0000236089.44566.62 16946668

[B88] Ohlemiller K. K. (2009). Mechanisms and genes in human strial presbycusis from animal models. *Brain Res.* 1277 70–83. 10.1016/j.brainres.2009.02.079 19285967 PMC2792931

[B89] Ohlemiller K. K. (2019). Mouse methods and models for studies in hearing. *J. Acoust. Soc. Am.* 146 3668–3680. 10.1121/1.5132550 31795658

[B90] Ohlemiller K. K. Dahl A. R. Gagnon P. M. (2010). Divergent aging characteristics in CBA/J and CBA/CaJ mouse cochleae. *J. Assoc. Res. Otolaryngol. JARO* 11 605–623. 10.1007/s10162-010-0228-1 20706857 PMC2975886

[B91] Ohlemiller K. K. Gagnon P. M. (2004). Cellular correlates of progressive hearing loss in 129S6/SvEv mice. *J. Comp. Neurol.* 469 377–390. 10.1002/cne.11011 14730589

[B92] Okano T. (2014). Immune system of the inner ear as a novel therapeutic target for sensorineural hearing loss. *Front. Pharmacol.* 5:205. 10.3389/fphar.2014.00205 25228882 PMC4151383

[B93] Overbeck G. W. Church M. W. (1992). Effects of tone burst frequency and intensity on the auditory brainstem response (ABR) from albino and pigmented rats. *Hear. Res.* 59 129–137. 10.1016/0378-5955(92)90110-9 1618705

[B94] Parthasarathy A. Kujawa S. G. (2018). Synaptopathy in the aging cochlea: characterizing early-neural deficits in auditory temporal envelope processing. *J. Neurosci.* 38 7108–7119. 10.1523/JNEUROSCI.3240-17.2018 29976623 PMC6596096

[B95] Patuzzi R. (2011). Ion flow in stria vascularis and the production and regulation of cochlear endolymph and the endolymphatic potential. *Hear. Res.* 277 4–19. 10.1016/j.heares.2011.01.010 21329750

[B96] Peeleman N. Verdoodt D. Ponsaerts P. Van Rompaey V. (2020). On the role of fibrocytes and the extracellular matrix in the physiology and pathophysiology of the spiral ligament. *Front. Neurol.* 11:580639. 10.3389/fneur.2020.580639 33193034 PMC7653186

[B97] Pilati N. Ison M. J. Barker M. Mulheran M. Large C. H. Forsythe I. D. (2012). Mechanisms contributing to central excitability changes during hearing loss. *Proc. Natl. Acad. Sci.U.S.A* 109 8292–8297. 10.1073/pnas.1116981109 22566618 PMC3361412

[B98] Schmittgen T. D. Livak K. J. (2008). Analyzing real-time PCR data by the comparative CT method. *Nat. Protoc.* 3 1101–1108. 10.1038/nprot.2008.73 18546601

[B99] Schuknecht H. F. Gacek M. R. (1993). Cochlear pathology in presbycusis. *Ann. Otol. Rhinol. Laryngol.* 102 1–16. 10.1177/00034894931020s101 8420477

[B100] Schulte B. A. Adams J. C. (1989). Immunohistochemical localization of vimentin in the gerbil inner ear. *J. Histochem. Cytochem.* 37 1787–1797. 10.1177/37.12.2685109 2685109

[B101] Schulte B. A. Schmiedt R. A. (1992). Lateral wall Na, K-ATPase and endocochlear potentials decline with age in quiet-reared gerbils. *Hear. Res.* 61 35–46. 10.1016/0378-5955(92)90034-K 1326507

[B102] Sha S.-H. Chen F.-Q. Schacht J. (2009). Activation of cell death pathways in the inner ear of the aging CBA/J mouse. *Hear. Res.* 254 92–99. 10.1016/j.heares.2009.04.019 19422898 PMC2749985

[B103] Sha S.-H. Kanicki A. Dootz G. Talaska A. E. Halsey K. Dolan D. (2008). Age-related auditory pathology in the CBA/J mouse. *Hear. Res.* 243 87–94. 10.1016/j.heares.2008.06.001 18573325 PMC2577824

[B104] Shin S.-A. Lyu A.-R. Jeong S.-H. Kim T. H. Park M. J. Park Y.-H. (2019). Acoustic trauma modulates cochlear blood flow and vasoactive factors in a rodent model of noise-induced hearing loss. *Int. J. Mol. Sci.* 20:5316. 10.3390/ijms20215316 31731459 PMC6862585

[B105] Soucek S. Michaels L. Frohlich A. (1986). Evidence for hair cell degeneration as the primary lesion in hearing loss of the elderly. *J. Otolaryngol.* 15 175–183. 3723657

[B106] Spicer S. S. Schulte B. A. (2002). Spiral ligament pathology in quiet-aged gerbils. *Hear. Res.* 172 172–185. 10.1016/S0378-5955(02)00581-6 12361880

[B107] Staecker H. Zheng Q. Y. (2001). Oxidative stress in aging in the C57B16/J mouse cochlea. *Acta. Otolaryngol.* 121 666–672. 10.1080/00016480152583593 11678164 PMC2862210

[B108] Stanojlovic M. Pallais J. P. Lee M. K. Kotz C. M. (2019). Pharmacological and chemogenetic orexin/hypocretin intervention ameliorates Hipp-dependent memory impairment in the A53T mice model of Parkinson’s disease. *Mol. Brain.* 12:87. 10.1186/s13041-019-0514-8 31666100 PMC6822428

[B109] Subramaniam M. Henderson D. Campo P. Spongr V. (1992). The effect of “conditioning” on hearing loss from a high frequency traumatic exposure. *Hear. Res.* 58 57–62. 10.1016/0378-5955(92)90008-b 1559906

[B110] Syka J. (2010). The fischer 344 rat as a model of presbycusis. *Hear. Res.* 264 70–78. 10.1016/j.heares.2009.11.003 19903514

[B111] Tan W. J. (2013). Noise-induced cochlear inflammation. *World J. Otorhinolaryngol.* 3:89. 10.5319/wjo.v3.i3.89 34549126

[B112] Tavanai E. Mohammadkhani G. (2017). Role of antioxidants in prevention of age-related hearing loss: a review of literature. *Eur. Arch. Otorhinolaryngol.* 274 1821–1834. 10.1007/s00405-016-4378-6 27858145

[B113] Tornabene S. V. Sato K. Pham L. Billings P. Keithley E. M. (2006). Immune cell recruitment following acoustic trauma. *Hear. Res.* 222 115–124. 10.1016/j.heares.2006.09.004 17081714

[B114] Trowe M.-O. Maier H. Schweizer M. Kispert A. (2008). Deafness in mice lacking the T-box transcription factor Tbx18 in otic fibrocytes. *Development* 135 1725–1734. 10.1242/dev.014043 18353863

[B115] Viana L. M. O’Malley J. T. Burgess B. J. Jones D. D. Oliveira C. A. C. P. Santos F. (2015). Cochlear neuropathy in human presbycusis: confocal analysis of hidden hearing loss in post-mortem tissue. *Hear. Res.* 327 78–88. 10.1016/j.heares.2015.04.014 26002688 PMC4554812

[B116] Wang J. Puel J.-L. (2020). Presbycusis: an update on cochlear mechanisms and therapies. *J. Clin. Med.* 9:218. 10.3390/jcm9010218 31947524 PMC7019248

[B117] Wang Q. Oyarzabal E. A. Song S. Wilson B. Santos J. H. Hong J. S. (2020). Locus coeruleus neurons are most sensitive to chronic neuroinflammation-induced neurodegeneration. *Brain Behav. Immun.* 87 359–368. 10.1016/j.bbi.2020.01.003 31923552 PMC7316605

[B118] Wang Y. Hirose K. Liberman M. C. (2002). Dynamics of noise-induced cellular injury and repair in the mouse cochlea. *J. Assoc. Res. Otolaryngol.* 3 248–268. 10.1007/s101620020028 12382101 PMC3202415

[B119] Wang Y. Qu Y. Chen X. Zhang P. Su D. Wang L. (2019). Effects of D-methionine in mice with noise-induced hearing loss mice. *J. Int. Med. Res.* 47 3874–3885. 10.1177/0300060519860679 31327277 PMC6726779

[B120] Watson N. Ding B. Zhu X. Frisina R. D. (2017). Chronic inflammation – inflammaging – in the ageing cochlea: a novel target for future presbycusis therapy. *Ageing Res. Rev.* 40 142–148. 10.1016/j.arr.2017.10.002 29017893 PMC5675822

[B121] World Health Organization (2021). *World Report on Hearing.* Geneva: World Health Organization.

[B122] Wu P. Z. Liberman L. D. Bennett K. de Gruttola V. O’Malley J. T. Liberman M. C. (2019). Primary neural degeneration in the human cochlea: evidence for hidden hearing loss in the aging ear. *Neuroscience* 407 8–20. 10.1016/j.neuroscience.2018.07.053 30099118 PMC6369025

[B123] Yamaguchi T. Nagashima R. Yoneyama M. Shiba T. Ogita K. (2014). Disruption of ion-trafficking system in the cochlear spiral ligament prior to permanent hearing loss induced by exposure to intense noise: possible involvement of 4-hydroxy-2-nonenal as a mediator of oxidative stress. *PLoS One* 9:e102133. 10.1371/journal.pone.0102133 25013956 PMC4094500

[B124] Yao W. Godfrey D. A. (1997). Densitometric evaluation of markers for cholinergic transmission in rat superior olivary complex. *Neurosci. Lett.* 229 21–24. 10.1016/s0304-3940(97)00400-x 9224792

[B125] Yoshida N. Hequembourg S. J. Atencio C. A. Rosowski J. J. Liberman M. C. (2000). Acoustic injury in mice: 129/SvEv is exceptionally resistant to noise-induced hearing loss. *Hear. Res.* 141 97–106. 10.1016/S0378-5955(99)00210-5 10713498

[B126] Yoshida N. Kristiansen A. Liberman M. C. (1999). Heat stress and protection from permanent acoustic injury in mice. *J. Neurosci. Off. J. Soc. Neurosci.* 19 10116–10124. 10.1523/JNEUROSCI.19-22-10116.1999 10559419 PMC6782949

[B127] Zheng Q. Y. Johnson K. R. Erway L. C. (1999). Assessment of hearing in 80 inbred strains of mice by ABR threshold analyses. *Hear. Res.* 130 94–107. 10.1016/s0378-5955(99)00003-9 10320101 PMC2855304

